# Directional Lighting-Based Deep Learning Models for Crack and Spalling Classification

**DOI:** 10.3390/jimaging11090288

**Published:** 2025-08-25

**Authors:** Sanjeetha Pennada, Jack McAlorum, Marcus Perry, Hamish Dow, Gordon Dobie

**Affiliations:** 1 Department of Civil & Environmental Engineering, University of Strathclyde, Glasgow G1 1XJ, UK; 2 Department of Electronic & Electrical Engineering, University of Strathclyde, Glasgow G1 1XJ, UK

**Keywords:** multi-channel neural network, transfer learning, stratified cross-validation, image fusion, hyperparameter tuning, structural health monitoring

## Abstract

External lighting is essential for autonomous inspections of concrete structures in low-light environments. However, previous studies have primarily relied on uniformly diffused lighting to illuminate images and faced challenges in detecting complex crack patterns. This paper proposes two novel algorithms that use directional lighting to classify concrete defects. The first method, named fused neural network, uses the maximum intensity pixel-level image fusion technique and selects the maximum intensity pixel values from all directional images for each pixel to generate a fused image. The second proposed method, named multi-channel neural network, generates a five-channel image, with each channel representing the grayscale version of images captured in the Right (R), Down (D), Left (L), Up (U), and Diffused (A) directions, respectively. The proposed multi-channel neural network model achieved the best performance, with accuracy, precision, recall, and F1 score of 96.6%, 96.3%, 97%, and 96.6%, respectively. It also outperformed the FusedNet and other models found in the literature, with no significant change in evaluation time. The results from this work have the potential to improve concrete crack classification in environments where external illumination is required. Future research focuses on extending the concepts of multi-channel and image fusion to white-box techniques.

## 1. Introduction

Early detection of damage through Structural Health Monitoring (SHM) is crucial in minimizing the costs associated with reconstruction and extending the service life of structures [1,2]. Surface defects such as cracks and spalling must be identified promptly, as they can negatively impact the occupants’ well-being and the structural integrity of the building. Cracking is a common defect in concrete civil infrastructure that can develop rapidly due to environmental conditions and material ageing. The initial stage of various diagnosis and inspection procedures for concrete structures is crack detection [3]. Spalling occurs when materials are ejected from the surface structure due to impact or internal stress. Moisture incursion is one of the primary causes of spalling in concrete. If left unidentified and untreated, it can lead to corroded reinforcements and a decline in structural durability [4]. It is essential to prioritize regular and accurate inspections to ensure the safety and longevity of concrete structures. Many industries rely on periodic inspections to monitor the condition of their structural assets [5].

Real-time non-destructive testing (NDT) methods enable engineers to continuously evaluate the state of the concrete structure without causing permanent damage [6]. The introduction of automation to visual inspection has great potential to be an efficient non-destructive evaluation (NDE) method for automatically detecting defects in concrete structures [7]. This method offers faster, safer, and more accurate results compared to the expensive, time-consuming, and labor-intensive conventional manual inspection techniques [8,9,10,11,12,13,14,15,16,17].

Manual inspections often suffer from high error rates due to human factors such as fatigue, lack of expertise, and subjective judgment. Studies have shown that error rates in manual inspections can be as high as 20–30%, with critical defects often overlooked or misidentified [18]. Automated inspection techniques have proven to be highly efficient, reducing the average time consumed for major activities by 57.85% compared to manual inspection methods. Additionally, they also reduce networking costs by an average of 51.67% and enhance output quality, thereby decreasing costs incurred for rework and scrap by 66.76% [19].

Automated defect detection can be separated into white-box and black-box techniques [17,20,21,22,23]. White-box techniques use mathematical operators and algorithms, such as edge detectors and thresholding; the black-box techniques employ machine learning and artificial neural networks [24,25]. Both methods have unique benefits that depend on the application, but generally, the black-box performs better for initial detection, whereas the white-box is more suited for pixel-level segmentation [26].

### 1.1. Review of Illumination Techniques and Concrete Defect Detection Algorithms

Despite the widespread use of illumination techniques to improve image capture in various industries [27,28,29], the impact of illumination variation on automated concrete inspections has not been extensively explored. In contrast, during concrete inspections, human inspectors frequently adjust the illumination using flashlights. Although some experiments have demonstrated the effectiveness of convolutional neural network (CNN) techniques in detecting defects under challenging lighting conditions [30,31,32]; however, these methods require a significant amount of training data. The process of acquiring and labeling such a dataset can be time-consuming and resource-intensive, which must be taken into consideration when implementing these techniques [33].

Numerous studies have demonstrated that brightness and lighting direction significantly influence crack detection accuracy, yet many neural network models often fail to fully utilize the advantage of directional lighting [34,35]. Ref. [30] focused on object detection in poor lighting conditions using YOLO (You Only Look Once) and faster region-based CNN (RCNN) deep learning models. These images were categorized based on lighting types, such as low, ambient, weak, object, and single, to analyze their impact on object detection. The results indicated that YOLO outperformed Faster RCNN. Ref. [31] explored low-light object detection by comparing various image enhancement algorithms and detection models but faced limitations in accurately detecting objects under these conditions, leading to lower detection performance. The Crack Image Analysis System in [36] computes geometric parameters of crack networks under uniform illumination, typically provided by a diffuse and evenly distributed light source. However, this reliance on uniform lighting may limit its accuracy in low-light conditions, reducing the visibility and contrast of crack features. Therefore, adapting the system for low-light conditions could enhance its robustness and reliability. Ref. [37] implemented the DSD-Net algorithm, combining Fast Fourier Transform (FFT) with a CNN. The frequency domain branch of the model uses FFT for spatial and frequency domain conversion, mitigating the impact of low brightness. The CNN coding branch extracts contextual information, and a feature fusion detection module enhances crack localization accuracy, making it effective for detection in low-light environments. However, the model faced challenges in detecting complex crack patterns and achieved an F1 score of 86.9%, indicating there is still room for improvement. Ref. [38] utilized directional lighting to illuminate the concrete surface that needs to be inspected in order to detect defects. However, a drawback of this method is the need for close positioning of the lights, leading to uneven illumination. Therefore, optimizing the directional lighting setup could improve visibility and addressing the uneven illumination could enhance crack detection.

Previous studies mainly focused on laboratory datasets, which do not account for the complexities and variations present in real-world scenarios, such as different illumination conditions and image scene environments. This limitation highlights the need for algorithms that can effectively handle the challenges posed by real-world crack images [39]. Therefore, there is a need to develop more robust and effective object detection algorithms specifically tailored for low-light environments.

In our previous work [40], we evaluated the potential of directional and angled lighting by training neural network models on each of the directionally lit datasets individually and demonstrated that angled and directional lighting can significantly improve the detection of concrete cracks in low-light environments compared to traditional diffused lighting. Ref. [17] explored the impact of angled and directional lighting on pixel-level segmentation of concrete cracks, leaving room for further exploration of directional lighting models for concrete defect classification. However, this study addresses the limitations of [16] by implementing directional lighting-based neural network models to classify cracks and spalling reliably and accurately on real-world data.

The conventional CNNs typically use three-channel inputs corresponding to an image’s RGB (Red, Green, and Blue). The state-of-the-art model (i.e., Zoubir’s model) used in this study utilizes a fine-tuned Visual Geometry Group (VGG16) architecture, which has been recognized as a state-of-the-art approach for defect classification in concrete structures. It achieved high performance metrics, including an F1 score of 97.38% for crack detection, demonstrating its effectiveness in accurately identifying and localizing defects (like cracks, spalling, and efflorescence) under diverse conditions. Therefore, in this study the model in [41] is trained and tested on three-channel diffused images.

In this study, traditional and Zoubir’s models are compared with two proposed models: fused CNN (FusedNet) and multi-channel CNN (MCNet), which utilizes the images captured in five lighting directions: Right (R), Down (D), Left (L), Up (U), and Diffused (A), respectively. The varied lighting directions create and cast shadows in the defects.

### 1.2. Research Contributions

There are two ways to address the problems: modifying the data to suit standard models (FusedNet) or modifying the models to suit the five-channel data (MCNet). This paper includes the implementation of both these models. The research contributions carried out are as follows:

1. Study the influence of lighting direction on the performance of automated defect detection algorithms.

2. Implementation of the novel FusedNet model that employs pixel-based maximum intensity image fusion technique to detect defects in concrete structures.

3. Development of the five-channel neural network model (MCNet) to effectively classify cracks (widths ranging from 0.07 mm to 0.3 mm) and spalling in concrete structures.

4. Investigate the importance and potentiality of directional lighting in both binary and multi-class image classification tasks utilizing FusedNet and MCNet.

5. Compare the performance of the FusedNet and MCNet models with the conventional (traditional) three-channel model and Zoubir’s model that utilize diffused images alone. All the models are implemented using hyperparameter tuning, the early stopping regularization technique, and a stratified five-fold cross-validation approach to avoid overfitting and improve overall robustness and generalization of the models.

6. Evaluate the performance of the proposed models on real-world concrete structures.

7. Investigate how increased exposure impacts pixel intensity in fused images and evaluate whether this impact is similar for fused and diffused images and assess the subsequent impact on the performance of neural network model.

## 2. Background

CNNs provide a robust object recognition and classification solution and have been integrated into structural damage detection methods [42]. They extract different levels of features, starting from basic edges and patterns to more complex object shapes and colors, leading to improved classification and segmentation. They have become widely adopted due to their ability to capture and utilize features from input data [43,44]. The majority of classification algorithms that use the CNN model follow two fundamental steps: first, they use convolutional layers to highlight the object’s key features, and then they employ fully connected (FC) layers to classify objects based on the output features of the convolutional step [45]. Some studies suggest that CNNs face challenges in identifying cracks that are narrower than 2 mm wide [46].

### 2.1. Limitations of CNNs in Structural Inspections

Researchers have proposed various CNN-based models for crack and spalling detection. For example, Pauly et al. [47] investigated the effect of network depth on crack classification, concluding that deeper networks are capable of learning more information, i.e., the deeper the networks are, the more they learn about detecting cracks. The study also found that networks trained on images from a specific location do not perform well when tested on images from a different location. Images were captured either manually or semi-automatically using smartphones, under ambient lighting conditions, leading to performance degradation (85.6% precision) due to variations in lighting across different locations.

Wang and Hu [48] developed a novel method for automatic pavement crack detection and classification, utilizing a CNN to detect cracks and principal component analysis to classify them into longitudinal, transverse, and alligator cracks. The study found that pavement images obtained outdoors have large illumination variations that can introduce challenges in classifying cracks from the background, and no specific techniques were proposed to address this drawback.

Dorafshan et al. [26] compared an AlexNet deep convolutional neural network model with edge detectors for image-based crack classification in concrete. The results were obtained using high-quality images taken under good lighting conditions, free from other environmental factors. The extension of these findings to real-world scenarios, where lighting conditions may be poor or uneven, is limited.

Dorafshan et al. [49] conducted a comparative study between fully trained and transfer learning modes using an AlexNet-based deep learning convolutional neural network for crack classification tasks in small Unmanned Aerial Systems (sUAS)-assisted structural inspections of concrete bridge decks and buildings. The study highlights the need for improved handling of poor lighting conditions, as along with image blurriness from sUAS vibrations and low-resolution cameras, as they can negatively impact crack classification accuracy, potentially misleading both human inspectors and conventional image-processing methods.

Park et al. [50] proposed a CNN architecture for patch-based crack detection in black box images, classifying road surface elements into three categories: crack, road marking, and intact regions. However, the study highlights that image-processing techniques for crack detection, including the proposed CNN, may not perform well under low lighting conditions, which can lead to inaccuracies and misclassifications due to reduced contrast and visibility of cracks.

All the studies mentioned above refer to crack detection and classification on concrete surfaces. There are very few studies for detecting and classifying spalling in concrete. Bai et al. [51] has developed three Mask R-CNNs to detect cracks and spalling in concrete structures. Yasmin et al. [52] proposed an encoder-decoder-based deep architecture for detecting and classifying the severity level of spalling. Mohammed Abdelkader et al. [53] combined advanced image processing techniques, including entropy-based segmentation and noise filtering, with machine learning models to accurately classify the spalling damage into severity levels based on area and depth. Hoang et al. [4] proposed an automated method for detecting concrete spalling using a piecewise linear stochastic gradient descent logistic regression model combined with image texture analysis. This method extracts texture features from concrete surface images to classify the image samples as spall or no spall under well-lit conditions. To date, no studies have addressed the identification of spalling under low-light conditions.

### 2.2. Addressing Limitations in Concrete Crack Detection

In the study of concrete crack detection, algorithms are often challenged by uncontrolled or low-light conditions. Jayanthi et al. [54] proposed an algorithm that improves crack detection in low-light conditions by first removing haze from the image using a contrast stretching method. It then converts the image to grayscale, applies thresholding, and utilizes morphological skeletonization for accurate detection of hairline cracks. This algorithm may struggle to differentiate cracks from background noise, especially in low-light images. Addressing these drawbacks is essential to enhance the effectiveness of the algorithm in accurately detecting cracks under challenging lighting conditions. Additional lighting can enhance crack visibility and reduce noise in images, leading to more precise detection.

Advanced image processing methods often assume uniform lighting conditions for detecting cracks in concrete structures, but they struggled to detect hairline cracks [55]. Therefore, exploring alternative geometrical illumination methods could further enhance the accuracy and efficacy of these algorithms. By addressing the challenges posed by low-light conditions and optimizing lighting techniques, concrete crack detection algorithms can be significantly improved, leading to better maintenance and safety of civil structures. This study uses geometrical illumination techniques, leveraging different lighting angles and directions to highlight small differences in texture and surface properties of concrete cracks.

Subsequent studies aimed to conduct crack detection in real-world settings but encountered challenges with illumination, obtaining clear images, and accurately identifying defects [56]. Additionally, the quality of crack detection is affected by various factors such as camera specifications, surface illumination, and environmental conditions, leading to unclear images and increased false positive rates [26]. In Padalkar et al. [57], it has been found that the height of illumination sources had a significant impact on crack detection accuracy, with higher placements resulting in improved outcomes and a reduction in false positives, suggesting that optimal lighting conditions are essential for effective crack detection in visual inspection systems.

The state-of-the-art automated inspections lack the following capabilities:

- Utilization of hardware with illumination techniques such as geometrical illumination (direction) to aid in automated defect detection/analysis.
- Analysis of the influence of lighting direction on the performance of automated defect detection algorithms.
- Analysis of directional lighting utilized defect detection algorithms for binary and multi-class image classification tasks.

This study aims to address these limitations in the current literature.

### 2.3. MobileNetV2 Neural Network Model

Popular CNN architectures like AlexNet, VGG16, InceptionV3, and ResNet50 are trained on large image datasets such as ImageNet [58]. These architectures have shown state-of-the-art performance for general image classification tasks. Compared to other CNNs, MobileNetV2-based CNNs have a smaller size and better computational efficiency without sacrificing performance [59]. Given its widespread use in civil engineering applications with a high success rate in identifying and categorizing cracks in concrete structures, it was the natural choice [60]. Therefore, no further exploration was undertaken to employ a different CNN architecture.

#### 2.3.1. Architecture

MobileNetV1 was developed based on the VGG architecture and incorporated convolutional layers to enhance accuracy. However, adding more layers led to gradient vanishing issues, which hindered the effectiveness of training. To address this, ResNet introduced the residual block, which enhanced information flow and mitigated the vanishing gradient problem. The skip connections in residual blocks allowed gradients to propagate more smoothly through the network during back-propagation, facilitating the training of deep neural networks [61].

**Figure 1:**
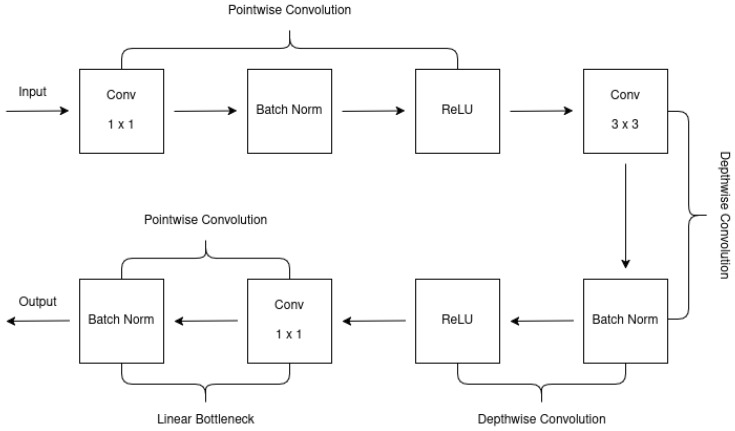
Inverted residual linear bottleneck taken from [61].

MobileNetV2 improved upon MobileNetV1 by incorporating the residual structure of ResNet and adding a linear bottleneck implementation. The linear bottleneck reduces computational complexity by simplifying convolution calculations, enhancing efficiency. Additionally, MobileNetV2 uses depth-wise separable convolutions, which split the convolution into depth-wise and point-wise operations, reducing parameters and computational cost. The inverted residual block in MobileNetV2 allows for better information flow and gradient propagation, improving the effectiveness of training. These innovations collectively enhance MobileNetV2’s performance, surpassing MobileNetV1 in both accuracy and efficiency, as demonstrated in Figure 1 and Table 1 taken from [61].

One way to transform features from N channels to M channels is by using a block with a specified stride (s) and expansion factor (t). This block includes a 1 × 1 convolutional layer followed by the depth-wise convolutional layer, with linear activation instead of nonlinear activation after the point-wise convolutional layer. Down-sampling can be achieved by adjusting the parameter ’s’ in the depth-wise convolutional layer [61]. MobileNetV2 utilizes convolutions and average pooling for processing the input data, with specific configurations denoted by parameters such as c (number of output channels) and n (number of repetitions). The overall network structure of MobileNetV2 is shown in Table 2 taken from [61].

#### 2.3.2. Transfer Learning with MobileNetV2

Deep learning models like MobileNetV2 have successfully used transfer learning and fine-tuning approaches to achieve accurate predictions in image classification problems [60]. Transfer learning involves using a pre-trained model trained on a large dataset like ImageNet [58] for image classification, where the learned features can be applied to various computer vision tasks. Fine-tuning is a technique where selected layers of a pre-trained model are unfrozen and trained together with the newly added classifier, allowing the model to adapt to a specific task or new dataset [62].

## 3. Methodology

The traditional MobileNetV2 model has three-channel input, which corresponds to the RGB (Red, Green, Blue) components of an image. In contrast, the two proposed models, FusedNet and MCNet, utilize images captured in five lighting directions to generate fused and five-channel images, with three- and five-channel inputs, respectively. The implementation of the three-channel FusedNet model is straightforward and similar to the traditional three-channel model but uses fused images instead. However, the MCNet model combines all the directional images to create a single five-channel Tagged Image File Format (TIFF) image, with each channel representing a specific lighting direction. TIFF is a versatile and widely used file format for storing high-quality images, making it ideal for multi-channel image analysis applications [63].

### 3.1. Overview

**Figure 2:**
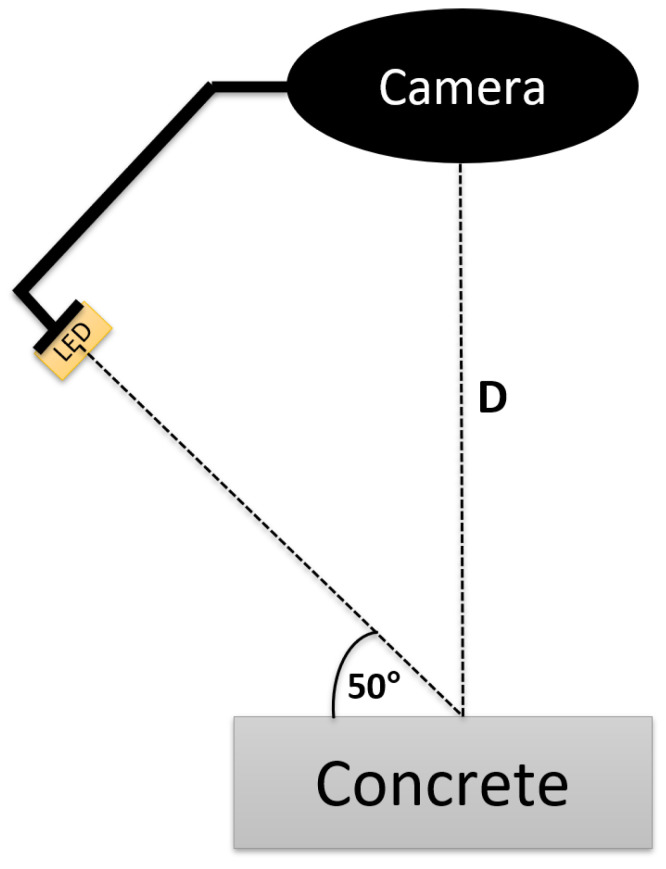
Specialized inspection equipment with single adjustable arm for a 50-degree lighting projection onto concrete surface taken from [20].

The inspection device shown in Figure 2 is used to capture images under directional lighting settings, utilizing a machine-vision camera surrounded by four adjustable arms, each fitted with RGB LED strips. These lighting arms are positioned at a 50-degree angle of incidence relative to the concrete surface [21,40]. Figure 2 illustrates this setup with a single arm, where D denotes the working distance.

### 3.2. Hardware and Settings

According to engineering standards, crack widths within the range of 0.1 mm to 0.3 mm in concrete structures should be identified for further action [64]. Therefore, the objective is to achieve a minimum spatial resolution of ≤0.1 mm/pixel during image capture.

**Figure 3:**
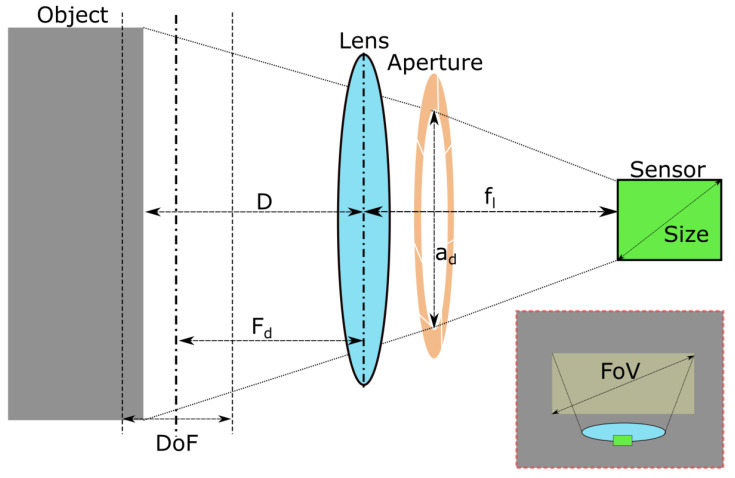
Illustration of variables during image capture. Light from an imaged object passes through the lens and aperture onto the camera sensor taken from [20].

An illustration of the image acquisition hardware is shown in Figure 3. In this work, a FLIR Blackfly 1” sensor machine vision camera was used [65]. It has an imaging resolution of 5472 × 3648 pixels and a focal length of $F_{l}$ = 8 mm. Together, these provided a feature resolution of ≤0.1 mm/pixel at a working distance of *D* = 350 mm, with a Field of View (FoV), of 574 mm × 383 mm [40]. For various imaging applications, the lightweight design of FLIR Blackfly® S cameras makes them a popular choice [65].

The depth of field, DoF, defines the distances at which objects remain in focus and is given by the following Equation (1):

(1) $$DoF=\frac{2\cdot F_{d}^{2}\cdot f_{n}\cdot C}{F_{l}^{2}},$$

where, $f_{n}=\frac{f_{l}}{a_{d}}$ indicates the f-number, which is the ratio of the focal length $f_{l}$ to the aperture diameter $a_{d}$. The variable *C* refers to the circle of confusion, a subjective threshold that determines an acceptable level of focus loss and is obtained by dividing the diagonal size of the camera sensor by 1500 [21].

Equation (1) shows that controlling the f-number, $f_{n}$, and focal distance, $F_{d}$, will vary the available DoF. $f_{n}$ is typically limited to the following values: 1.4, 2, 2.8, 4, 5.6, 8, 16. For a constant D and $F_{d}$, there is a trade-off when choosing $f_{n}$: a lower $f_{n}$ grants fast speed of image capture (exposure rate) and low diffraction of the image at the cost of blurred corners and small DoF. Diffraction causes a loss of sharpness in an image due to interfering light waves. For consistency, all images captured in this work were taken at a constant $f_{n}$ of 8 and $F_{d}$ of 250. This provided a middle ground for avoiding diffraction and blurred corners, with sufficient DoF = 172 mm, covering D from 200 to 350 mm.

The imaging hardware employed in this study features a configurable lighting system capable of capturing images under both multi-angle and multi-directional illumination. In our earlier work [20,40], we systematically investigated various lighting angles and identified 50 degrees as the optimal configuration for crack detection. Therefore, all the images captured in the present study were conducted using the 50-degree lighting angle to ensure consistency. Although the hardware can operate under a wider range of lighting conditions, a full multi-angle analysis was not repeated here, as it has already been reported in [20,40] and lies beyond the scope of this investigation. This approach allowed the current work to focus on leveraging the advantages of directional lighting for improved detection of cracks and spalling, particularly in low-light environments.

#### 3.2.1. Distortion Coefficient

All camera lenses have distortion coefficients that can be calculated and compensated for checkerboard calibrations. In this work, numerous images of a checkerboard pattern at various distances and angles were captured. The pattern was automatically identified and used to determine the coefficients required to reconstruct a straight checkerboard pattern. These coefficients were then used to correct for distortion on every image captured throughout this work.

#### 3.2.2. Exposure and White Balance

White color balance and exposure settings can be automatically calculated and adjusted by a camera’s on-board algorithms, but this is a slow process relative to the rapid changes in lighting conditions. Having preset values for exposure and white-balance for each lighting condition ensures exposure changes are fast and consistent. For this work, it was assumed there is no ambient lighting interference. The exposure is only dependent on the illumination used.

A sensitivity study was conducted to find the camera’s recommended exposure and white-balance settings across all lighting configurations. The recommended exposure was found using the in-built auto-exposure algorithm of the camera [65]. In summary, this algorithm compares a histogram of pixel brightness to an optimal mean and variance to find the optimal exposure. While white-balance settings remained fairly constant, exposure settings were found to be dependent on lighting angle when images were illuminated from a single direction. The exposure setting required for a lighting angle $\theta_{L}$ is:

(2) $$E_{\theta_{L}}=\left[2-\left(\frac{50-\theta_{L}}{50}\right)\right]\cdot E_{50}$$

where $E_{50}$ is the exposure setting required during diffused lighting at a 50° illumination angle (calibrated once at the beginning of a scan). Essentially, it was found that the auto-exposure for directional lighting was double the diffused exposure at a 50-degree lighting angle, and varied linearly with angle. This means a single auto-exposure calculation can be performed at 50 degrees during diffused lighting, and all other exposures can be computed using Equation (2).

### 3.3. Dataset Description and Pre-Processing

The initial dataset included five high-resolution images captured under directional lighting conditions at an optimal angle of 50 degrees in the laboratory. The dataset consisted of damaged, reinforced concrete slabs measuring 500 mm × 500 mm × 10 mm. The cracks were generated by applying forces at the edges, with widths ranging from 0.07 mm to 0.3 mm.

**Figure 4:**
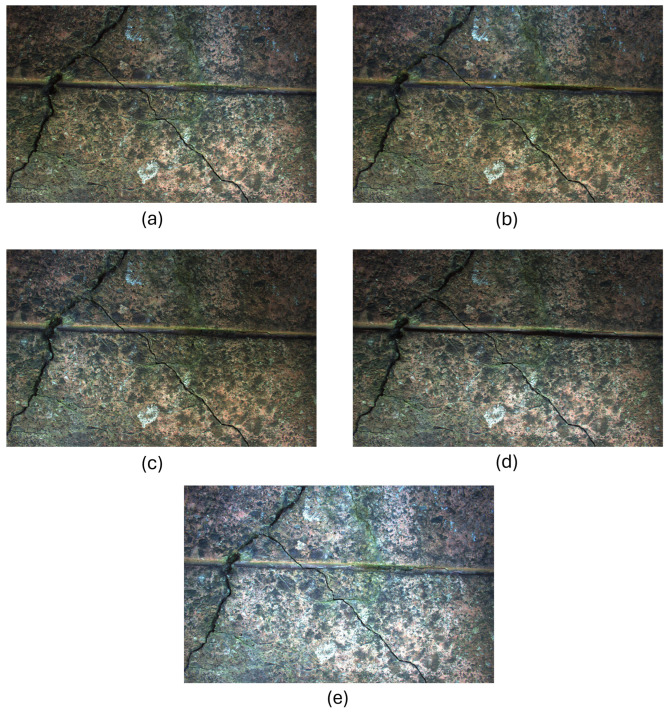
Captured images in real-world by projecting light from (**a**) right (R), (**b**) down (D), (**c**) left (L), (**d**) up (U), and (**e**) diffused (A) directions, respectively.

**Figure 5:**
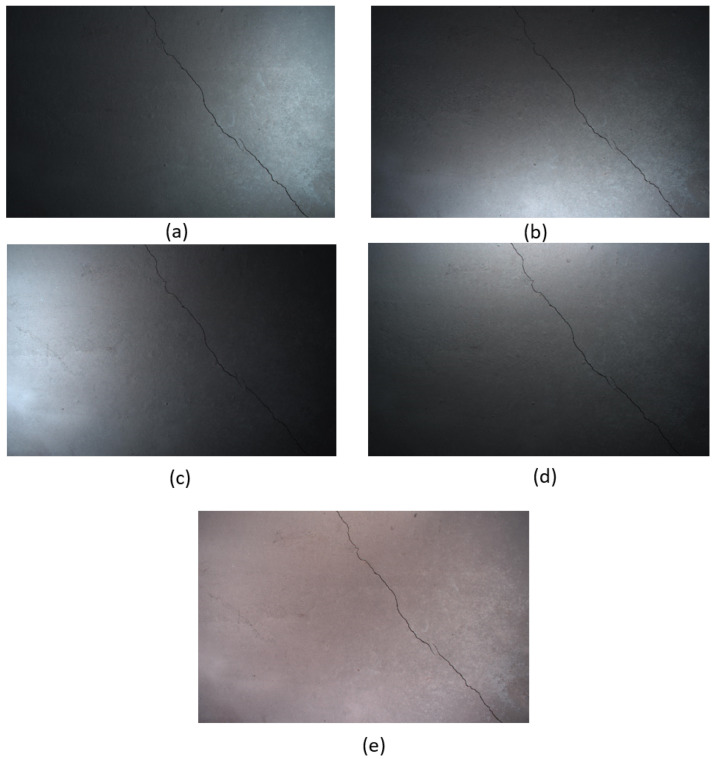
Captured images in laboratory by projecting light from (**a**) right (R), (**b**) down (D), (**c**) left (L), (**d**) up (U), and (**e**) diffused(A) directions, respectively.

The remaining four images that constitute the real-world dataset were formed from a range of concrete structures from different locations across the UK under normal environmental conditions. This ensures diversity in the data. Therefore, there are a total of 45 samples—9 images captured in R, D, L, U, and A directions, respectively, as shown in Figure 4 and Figure 5.

As Mobilenetv2 requires an image input size of 224 × 224 pixels, the captured images (measuring 5429 × 3458 pixels) were cropped into multiple sub-images, each measuring 224 × 224 pixels. These cropped sub-images undergo further pre-processing to form five-channel and fused images before being fed into the proposed models, explained in detail in the following subsections. All experiments were conducted on the original captured images without data augmentation.

### 3.4. Fused Image Generation

The maximum intensity pixel-level image fusion method [66] is widely utilized in the medical field to merge information from various modalities like Computed Tomography, Magnetic Resonance Imaging, Functional Magnetic Resonance Imaging, Single-Photon Emission Computed Tomography, and Positron Emission Tomography scans. This technique enhances the quality and clarity of medical images by selecting the pixel with the highest intensity from each corresponding location in multiple input images, each captured under different lighting conditions. It provides more comprehensive information for disease diagnosis and visualization [67,68,69]. However, this method has been largely overlooked in civil engineering applications.

**Figure 6:**
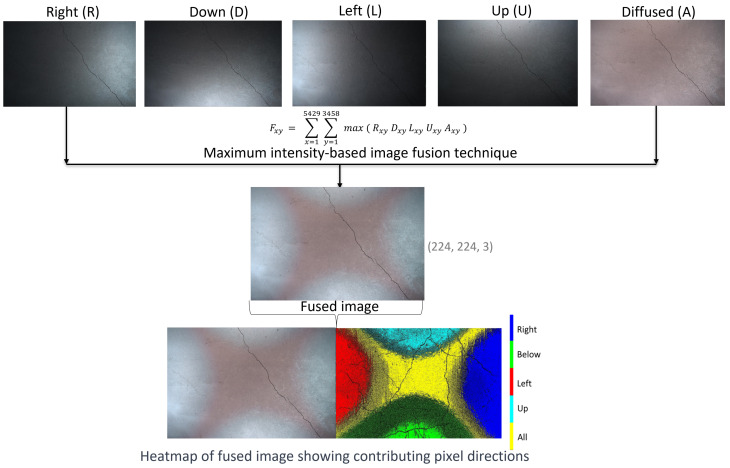
The images are captured in R, D, L, U, and A directions, respectively. These images are fused into a single image using the maximum-intensity image fusion technique. This involved comparing the pixel values from the five images at each location and selecting the maximum value. The heatmap of the resulting fused image indicates the contributing pixels from each direction.

The maximum intensity image fusion technique is used to obtain a single fused image from the five images captured under directional lighting conditions. This involves comparing the pixel values of corresponding pixels from the five directional images at each pixel position (x,y). The maximum value among the five input images is selected for each pixel. This process is repeated for each pixel across all five input images to generate the final fused image, as shown in Figure 6. As a result, the brightest pixel values are selected from each input image, and the heatmap clearly indicates the contributing pixels from each direction. The maximum intensity fusion technique for the directional images can be expressed using Equation (3).

(3) $$F_{xy}=\sum_{x=1}^{5429}\sum_{y=1}^{3458}\max\left(R_{xy}\cdot D_{xy}\cdot L_{xy}\cdot U_{xy}\cdot A_{xy}\right)$$

where the variables are as follows:

- $F_{xy}$ represents the pixel intensity value at position (x,y) in the output fused image F.
- $R_{xy}$, $D_{xy}$, $L_{xy}$, $U_{xy}$, and $A_{xy}$ are the pixel intensity values at position (x,y) in five directional input images R, D, L, U, and A, respectively.

### 3.5. Generation of a Five-Channel Image

**Figure 7:**
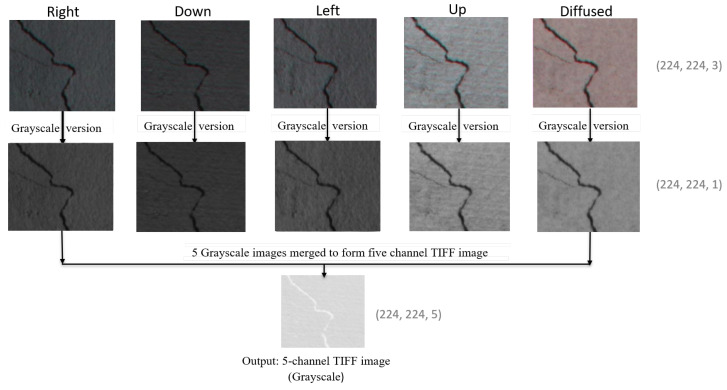
Generation of the five-channel TIFF image. The three-channel (RGB) color images (224, 224, 3) are captured in the right, down, left, up, and diffused directions (**top row**). These five images are further converted to one-channel grayscale images (224, 224, 1) (**second row**). These images are merged together to form a single five-channel TIFF image of size (224, 224, 5) (**bottom row**).

When using the MobileNetV2 architecture, an input image of size (224, 224, 3) with three color channels for red, green, and blue is required. However, the proposed MCNet model utilizes a five-channel TIFF input image. To generate a five-channel TIFF image, the initial step involves converting the original three-channel images captured under different lighting directions (R, D, L, U, A), into grayscale images (224, 224, 1), i.e., images of size 224 × 224 pixels with single gray channel. These grayscale images are then stacked together along the third dimension to form a single five-channel image (224, 224, 5), i.e., each grayscale image becomes a separate channel in the new multi-channel image. Therefore, each grayscale image corresponds to one channel, allowing the combined image to capture information from all five lighting directions. Finally, the resulting multi-channel image is saved in TIFF format, denoted by the .tiff extension, as illustrated in Figure 7. Since the five-channel generated images are of very high quality and contain image information of all five images, they are stored in TIFF format. This format is particularly suitable for the modified MobileNetV2 models, which expect an input image with dimensions of (224, 224, 5).

### 3.6. Dataset Structure Using Stratified k-Fold Cross-Validation

**Figure 8:**
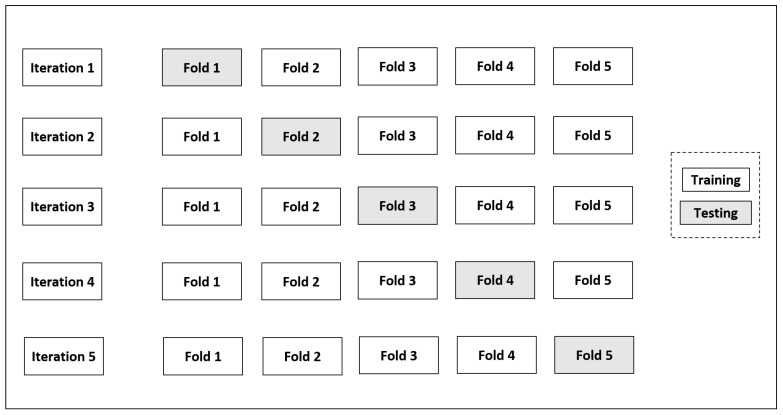
Stratified five-fold cross-validation: The gray boxes represent the folds used for testing the model, while the plain boxes represent the folds used for training the model.

To train the model, the data is split using stratified k-fold cross-validation (SKCV). The k value is typically chosen as five or ten for balancing variance and bias [70,71]. It divides the entire dataset into ‘k’ equal-sized subsets, or “folds,” ensuring that each fold contains nearly the same percentage of samples from both minority and majority classes. The process consists of ‘k’ iterations, each using ‘k-1’ folds for training the model and one fold for testing the performance of the model [71,72,73]. Performance metrics such as accuracy, precision, F1 score, recall, and MCC are calculated on the test fold for each iteration. After all the ‘k’ iterations are complete, the average performance across all iterations is calculated using equations described in Section 4.5. This provides a more reliable estimate of how well the model will perform on new, unseen data. Repeating the process ‘k’ times with different training and test fold combinations reduces the performance estimate variance. A stratified five-fold cross-validation technique is implemented in this study, where one subset was selected as the test set in every iteration, while the remaining four were used for training the model as shown in Figure 8.

### 3.7. Hyperparameter Tuning and Regularization

Hyperparameter tuning refers to the process of optimizing the hyperparameters of a machine learning model to improve its performance. They are settings that are external to the model and cannot be learned from the data, for example, batch size and learning rate. Batch size determines the number of samples processed before updating the model, and learning rate controls the step size during optimization [74]. The goal of hyperparameter tuning is to find the best combination of these parameters that results in the most accurate and efficient model for a given dataset [75].

Early stopping is a regularization technique to stop the training process before the model becomes too complex and starts to overfit the training data. By tracking accuracy on the validation set instead of the test set, it allows for finding the optimal hyperparameter values without overfitting. This strategy ensures a higher level of generalization and helps in reducing bias while increasing variance, ultimately improving the performance of the model. This approach not only reduces training time but also ensures better out-of-sample performance. Once training stops, the model with the best validation performance is selected for use [76].

All of the models utilized stratified five-fold cross-validation, hyperparameter tuning, and early stopping regularization techniques to prevent overfitting. The hyperparameter tuning was performed using learning rates of 0.1, 0.01, and 0.001, and batch sizes of 16, 32, and 64. The number of epochs was set to 100 with early stopping criteria.

## 4. Testing Methodology

### 4.1. Traditional MobileNetV2 Model

**Figure 9:**
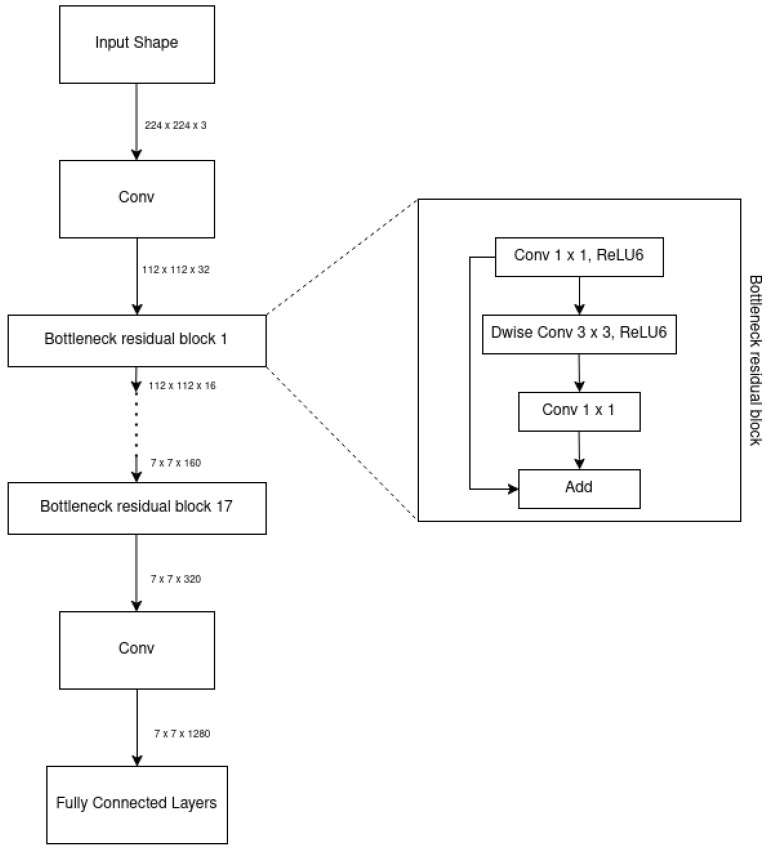
Architecture of the traditional three-channel MobileNetV2 model.

MobileNetV2 is a network architecture with 19 layers, designed for feature extraction and classification. The conventional MobileNetV2 model is shown in Figure 9. The overall network structure of MobileNetV2 is shown in Table 2.

### 4.2. Implementation of the FusedNet Model

**Figure 10:**
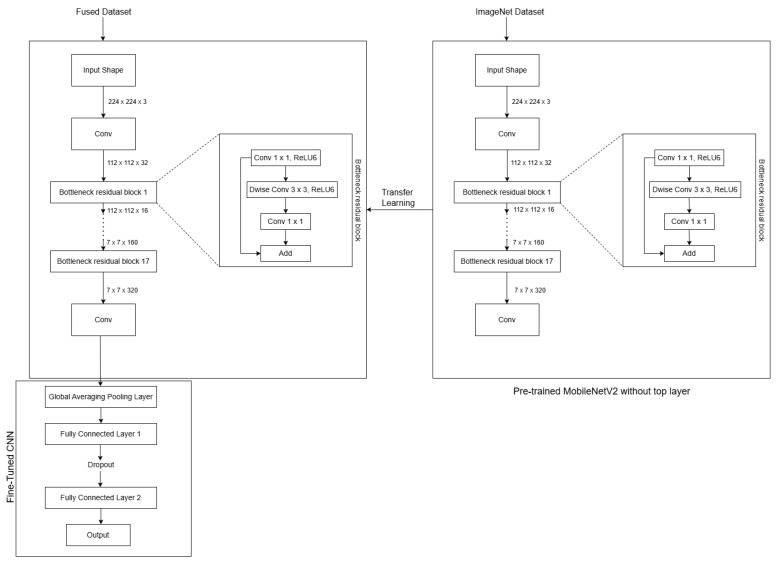
Architecture of FusedNet model.

The fused images are generated by fusing all the directional images using the maximum intensity image fusion technique as explained in Section 3.4. The architecture of FusedNet is shown in Figure 10.

FusedNet is built upon the pre-trained MobileNetV2 model and was further fine-tuned to adapt it to the new task. A custom sequential model is built on top of the base MobileNetV2, featuring two Dense layers with ReLU activation and a final Dense layer with either a sigmoid activation function for binary classification or softmax for multi-class image classification. The model is compiled with the Adam optimizer and uses the binary cross-entropy loss function for binary classification and the sparse categorical cross-entropy loss function for multi-class image classification.

### 4.3. Implementation of the MCNet Model

The MCNet model was developed to enhance defect classification in concrete structures by utilizing a customized five-channel input derived from grayscale images captured under various lighting conditions, as explained in Section 3.5. Table 3 shows that the three-channel models have 32 filters of size 3 × 3 kernel, resulting in a total of 288 weights. With three channels, the total number of weights is 864. In the case of the five-channel models, the number of filters, filter size, and number of weights per filter are the same. However, with five channels, the total number of weights becomes 1440. Therefore, the number of trainable parameters for the three-channel and five-channel MobileNetV2 models are 864 and 1440, respectively. This highlights the impact of the number of channels on the total trainable parameters in the first convolutional layer of the MobielNetV2 model.

Expanding on this knowledge, our approach takes advantage of the pre-trained MobileNetV2 model by transferring all its weights to the corresponding layers of the MCNet model, except the first convolutional layer. In order to accommodate the additional two channels in the first convolutional layer, we calculate their weight values by averaging the weights of the existing channels. This step allows us to extend the capability of the model to handle five-channel input while still benefiting from the pre-existing weights for the initial three channels. This approach preserves the spatial structure of the filters while producing a luminance-based, color-agnostic representation suitable for our grayscale directional-lighting images. This weight-initialization strategy is based on the assumption that low-level filters primarily detect edges, corners, and textures, which are largely independent of color. By averaging the original weights, we retain these spatial patterns, allowing the new channels to provide meaningful feature extraction.

Transfer learning and fine-tuning techniques are used to optimize the performance of the model in classifying the five-channel dataset for binary and multi-class image classification tasks. This involves utilizing the pre-trained weights of the MobileNetV2 model and fine-tuning them to adapt to the specific characteristics of our new five-channel dataset, enabling the model to efficiently learn the relevant features. The low-level filters are expected to adapt during training to capture lighting-specific shading variations relevant to defect classification.

**Figure 11:**
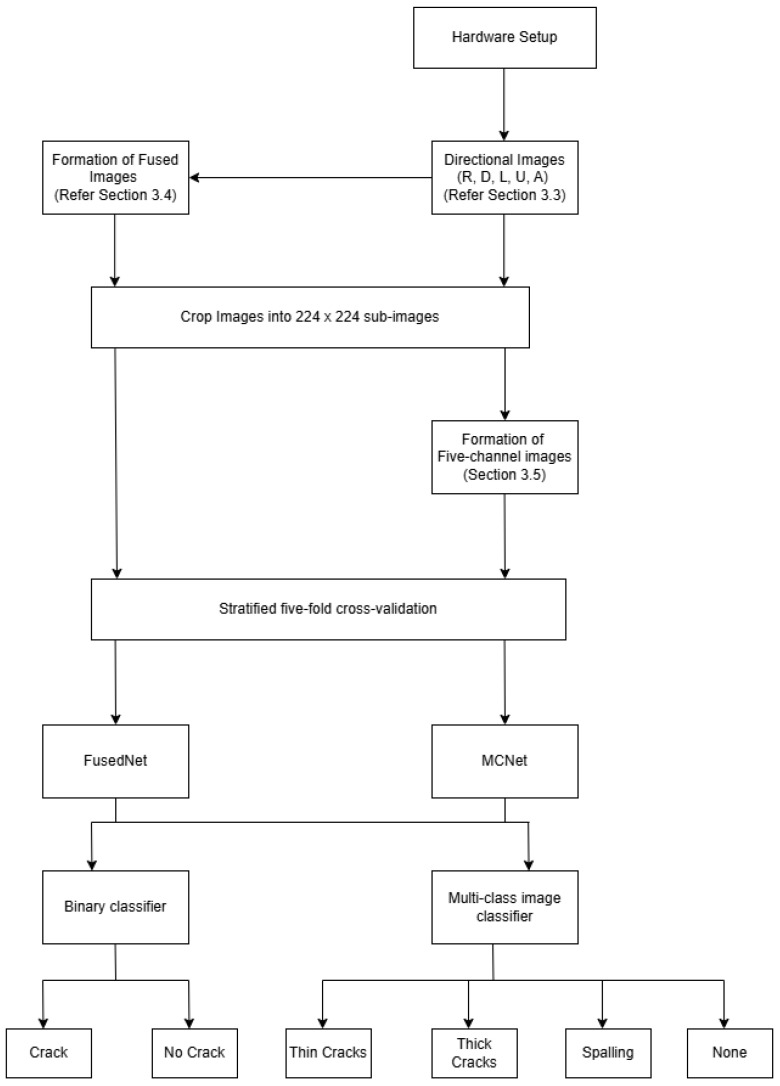
Step-by-step implementation of FusedNet and MCNet models for binary and multi-class image classification.

The step-by-step implementation of the MCNet model is elaborated in detail in the following section. The block diagram in Figure 11 showcases the step-by-step implementation of FusedNet and MCNet models for binary and multi-class image classification. Each block represents a distinct stage or operation involved in the process, demonstrating the logical flow of tasks.

#### 4.3.1. Five-Channel Dataset Preparation

A five-channel dataset was prepared to align with the requirements of the MCNet model. A well-balanced dataset was created for training and testing neural network models. For binary classification, the labels were positive (crack-1) and negative (no crack-0). For multi-class image classification, the labels were thin crack-0, thick crack-1, spalling-2, and none-3.

#### 4.3.2. Five-Channel MobileNetV2 (MCNet) Model Architecture

**Figure 12:**
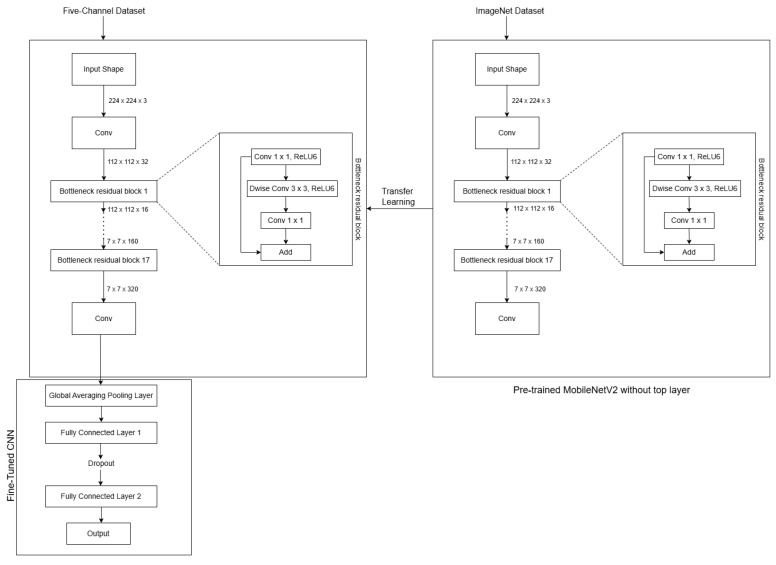
Architecture of five-channel MobileNetV2 (MCNet) model.

Figure 12 illustrates the architecture of the MCNet model. MCNet was built upon the MobileNetV2 architecture, which was originally trained on ImageNet. During fine-tuning, the weights of the pre-trained model are adjusted through training to accommodate the new five-channel dataset. To preserve the generalization capabilities of the pre-trained model and prevent overfitting, the early layers were frozen while the later layers were made trainable. Specifically, only the weights of the last six layers are updated during training. This strategy enhances the robustness and accuracy of the model in image classification tasks.

A custom sequential model is built on top of the base MobileNetV2, featuring two Dense layers with ReLU activation, and a final Dense layer with either a sigmoid activation function for binary classification or softmax for multi-class classification. The model is compiled with the Adam optimizer and uses the binary cross-entropy loss function for binary classification and the sparse categorical cross-entropy loss function for multi-class classification. The cross-entropy loss functions are utilized without any modification. The five directional channels are processed jointly, and during back-propagation, all channels contribute equally to the parameter updates with no channel-specific weighting.

#### 4.3.3. Transfer Learning and Fine-Tuning

The pre-trained MobileNetV2 model is used in transfer learning to capture low-level patterns and meaningful representations from the images, leveraging the knowledge it has acquired from the original task it was trained on. This makes it easier to recognize various generic features in concrete structure images. In fine-tuning, the pre-trained model’s weights are updated during training to adapt it to the new five-channel dataset. To maintain the generalization power of the pre-trained model and avoid overfitting, the early layers are frozen while the later layers are made trainable, as shown in Figure 12. This approach maximizes the utilization of the pre-trained model’s knowledge and enhances training efficiency, resulting in a more robust and accurate image classification solution.

### 4.4. Model Training

For the binary classification models, a total of 1338 images were utilized, while for the multi-class image classification task, a dataset of 525 images was used. A stratified five-fold cross-validation technique was employed throughout the study. In each iteration, one fold was used as the testing set, while the remaining four folds were used to train the model. The performance of the models was evaluated using various metrics, including accuracy, precision, recall, F1 score, and Matthews correlation coefficient (MCC).

### 4.5. Benchmarking and Performance Evaluation

Hyperparameter tuning is conducted to identify the optimal learning rate and batch size, ensuring the most accurate and efficient model for the given dataset. The models also incorporate early stopping to prevent overfitting and employ five-fold cross-validation to ensure robust performance on unseen data. Subsequently, the trained model is employed to make predictions on the testing dataset.

From the confusion matrix, the values for true positive (TP), true negative (TN), false positive (FP), and false negative (FN) are calculated. These values represent the correctly and incorrectly classified cases. To assess the model’s performance, various evaluation metrics shown in Table 4 and Table 5 are computed based on TP, TN, FP, and FN values. The model’s performance is evaluated based on accuracy, precision, recall, F1 score, and Matthew’s correlation coefficient (MCC) [77]. To determine these metrics, the precision, recall or true positive rate, accuracy, F1 score, and MCC are calculated for each of the five folds of the model and then averaged using Equations (4)–(8).

(4) $$\mathrm{Overall}\;\mathrm{Precision}=\frac{1}{k}\sum_{i=1}^{k}{\mathrm{Pre}}_{i}$$

(5) $$\mathrm{Overall}\;\mathrm{Recall}=\frac{1}{k}\sum_{i=1}^{k}{\mathrm{Rec}}_{i}$$

(6) $$\mathrm{Overall}\;\mathrm{Accuracy}=\frac{1}{k}\sum_{i=1}^{k}{\mathrm{Acc}}_{i}$$

(7) $$\mathrm{Overall}\;F1\;\mathrm{score}=\frac{1}{k}\sum_{i=1}^{k}{F1}_{i}$$

(8) $$\mathrm{Overall}\;\mathrm{MCC}=\frac{1}{k}\sum_{i=1}^{k}{\mathrm{MCC}}_{i}$$

where the variables are as follows:

i indicates the range of folds, which vary from 1 to k;

k indicates the total number of folds in the cross-validation;

Pre indicates precision;

Rec indicates recall;

Acc indicates the accuracy;

F1 indicates F1 score;

MCC indicates Matthew’s correlation coefficient.

## 5. Results and Discussion

### 5.1. Potential of Directional Lighting

The primary aim of this analysis was to assess how lighting direction affects the performance of the neural network model. Therefore, the MobileNetV2 neural network model underwent training and tested these directionally-lit images, covering nine samples across five directions (9 samples × 5 directions = 45 images).

**Figure 13:**
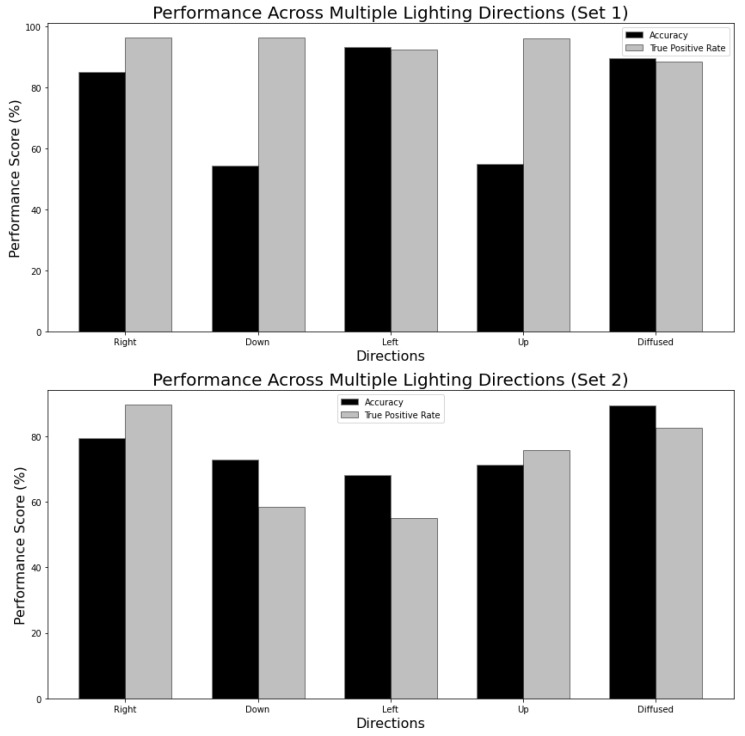
Performance across multiple lighting directions.

The performance of the trained neural network model in different lighting directions was investigated using two distinct sets. A detailed analysis of Sets 1 and 2 showed trends in the accuracy as shown in Figure 13. In Set 1, the model trained on images illuminated from the left direction achieved the highest accuracy of 93.1%. Also, the model trained on images illuminated from the right and down directions resulted in a higher true positive rate of 96.2%.

Set 2 achieved the highest accuracy of 89.4% when trained and tested on diffused images and the highest true positive rate of 89.7% when trained and tested on the images captured in the right direction. This shows that in Set 2, diffused lighting is more favorable for overall accuracy, while identification of positive instances is best achieved with lighting from the right direction.

The difference in the lighting orientation for accuracy and TPR emphasizes the possibility of optimizing directional lighting. In particular, for Set 1, the model shows good performance when trained on images lit from the left direction, whereas for Set 2, optimal performance is achieved with diffused lighting. These results highlight the potential of directional lighting. The choice of the most suitable lighting direction impacts the accuracy of the model and its TPR. In practical terms, optimizing the lighting direction conditions can improve the robustness of crack detection models in real-world scenarios. The potential of directional lighting in comparison to diffused lighting is evident, yet its full utilization requires modifications or implementation of new algorithms that incorporate directional lighting as discussed in Section 4.2 and Section 4.3.

### 5.2. Traditional MobileNetV2 vs. Zoubir vs. FusedNet vs. MCNet Models for Binary Crack Classification

**Figure 14:**
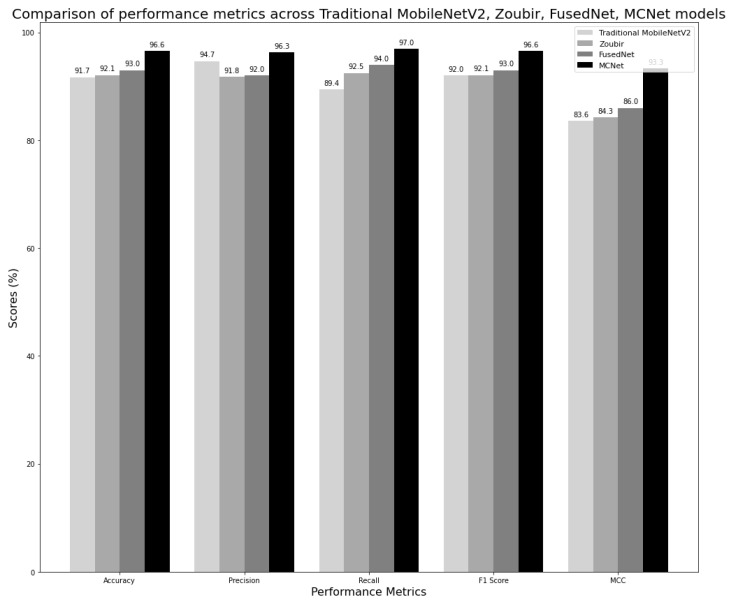
Comparison of classification metrics across Traditional MobileNetV2, Zoubir, FusedNet, and MCNet models.

For each of the three models, the specific hyperparameter values that yielded the best performance were identified. These optimal hyperparameter values were further used in the implementation of their corresponding multi-class image classification models. The Zoubir model, similar to the traditional model, is trained and evaluated on diffused images. Figure 14 shows a comparison of two proposed models, FusedNet and MCNet, with other models in the literature, which are the traditional and Zoubir models. The models were evaluated based on classification metrics including accuracy, precision, recall, F1 score, and MCC.

To mitigate the risk of overfitting, we employed a five-fold cross-validation approach (Section 3.6). This ensures that the model is trained and tested on multiple (five, here) splits of the data, providing a robust estimate of its generalization performance. Also, hyperparameter tuning was performed using learning rates of 0.1, 0.01, and 0.001, and batch sizes of 16, 32, and 64, while the number of epochs was capped at 100 with early stopping criteria applied (Section 3.7). Despite the relatively small dataset (e.g., 1338 images for binary classification), the combination of cross-validation, hyperparameter tuning, and early stopping ensures that the MCNet model learns meaningful features from the data without memorizing it, despite the increased number of parameters in the five-channel architecture.

**Figure 15:**
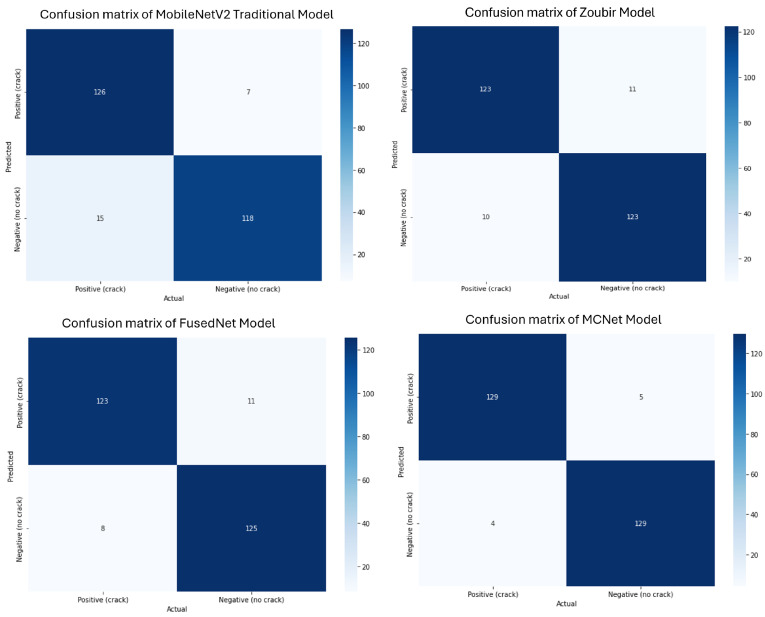
Confusion matrices of Traditional MobileNetV2, Zoubir, FusedNet, and MCNet models for binary crack classification.

All models achieved optimal performance with hyperparameter values set to 100 epochs (early stopping criteria), a batch size of 32, and a learning rate of 0.001. The comparison of the best-performing models, along with their confusion matrices, is shown in Figure 15. The reported performance metrics, including accuracy, precision, recall, and F1 score, are presented as the average values across all folds in the stratified five-fold cross-validation. This approach provides a more robust and representative assessment of model performance, reducing the influence of any single fold and accounting for variability in the dataset.

FusedNet achieved a 1.3% higher accuracy, a 4.6% increase in recall, a 1% better F1 score, and a 2.4% improvement in MCC compared to the traditional MobileNetV2 model. Although FusedNet’s precision is 2.7% lower than that of Traditional MobileNetV2, all the other metrics indicate that FusedNet is more effective overall, particularly in identifying actual crack instances. The significant boost in recall demonstrates the capability of FusedNet in detecting a higher number of true positives, thereby reducing the risk of missing any cracks. The improved F1 score shows that FusedNet maintains a good balance between precision and recall, ensuring both false positives and false negatives are well-identified. The higher MCC value further validates the overall robustness and reliability of FusedNet in crack classification. Despite the slight decrease in precision, which implies that FusedNet has a marginally higher rate of false positives, the FusedNet’s improved recall is crucial, as missing a crack could have severe consequences in civil engineering. This prioritizes the identification of all potential cracks, even if it means tolerating a few false alarms.

When compared to Zoubir, FusedNet shows a slight edge in performance. It has a 1% higher accuracy and a 1.5% better recall, indicating a greater capability to correctly identify and detect cracks. FusedNet also has a 0.2% higher precision, reflecting a slightly lower rate of false positives. Notably, it has a 0.9% higher F1 score and 1.7% higher MCC than Zoubir’s model, underscoring FusedNet’s overall robustness and reliability in crack detection. Overall, the higher accuracy, recall, F1 score, and MCC of FusedNet compared to Zoubir highlight its superior performance and effectiveness in detecting and classifying cracks. The use of fused images, which involves selecting the maximum intensity pixel value from a directionally lit dataset, notably improves crack visibility within the FusedNet model. This underscores the importance of directional lighting in enhancing contrast and clarity, thereby strengthening FusedNet’s ability to accurately detect and classify cracks.

In the comparison between FusedNet and MCNet for crack classification, MCNet consistently demonstrated superior performance across various metrics. MCNet achieved an accuracy of 96.6%, surpassing FusedNet by 3.6%, indicating its ability to classify a higher percentage of crack instances correctly. Moreover, MCNet exhibited higher precision at 96.3%, outperforming FusedNet by 4.3%, which signifies its lower rate of false positives and better precision in identifying actual crack instances. In terms of recall, MCNet achieved 97.0%, which is 3% higher than FusedNet’s recall of 94.0%, highlighting MCNet’s superior capability to detect more true positive crack instances. Additionally, MCNet achieved a higher F1 score of 96.6%, compared to FusedNet’s F1 score of 93.0%, indicating that MCNet showed a better overall balance between precision and recall. When considering the Matthews Correlation Coefficient (MCC), which provides a comprehensive measure of model performance accounting for true and false positives and negatives, MCNet significantly outperformed FusedNet by 7.3%.

While both FusedNet and MCNet utilize directional lighting, MCNet adopts a multi-channel neural network approach that incorporates features from all directional images to enhance crack detection. The confusion matrices of all the models were shown in Figure 15. The confusion matrix helps in understanding how well the proposed models outperformed the existing models in the literature in detecting and classifying cracks.

**Figure 16:**
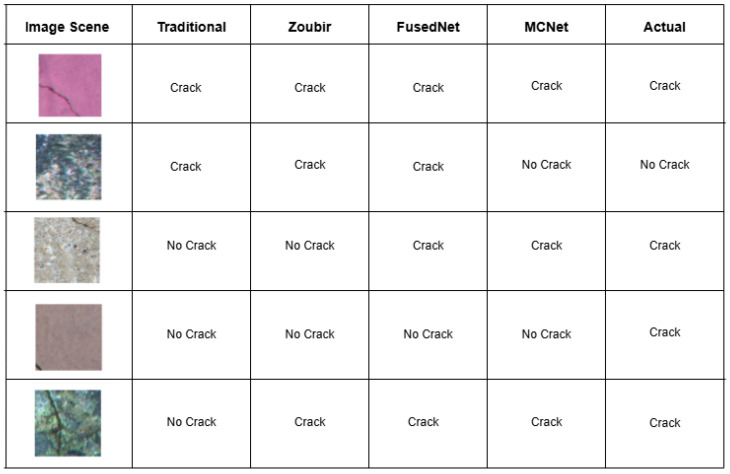
Model comparison for binary image classification on various samples.

Figure 16 shows qualitative results obtained when four trained models are tested on various samples. When the crack is relatively thick and runs through the center (Sample 1), all models successfully classified it with high confidence. However, when the crack is located near the extremities, such as along the edges (Sample 4), all models failed to detect it. When the crack is near but not at the edges (Sample 3), both proposed models were able to detect it. This suggests that directional lighting casts shadows in the defects, and help the models in detecting cracks more effectively compared to the traditional and Zoubir’s models. On uneven surfaces where the background is unclear, such as when algae are present (Sample 2), all models except MCNet mistakenly identified the algae as cracks. Additionally, when the sample has algae and the crack is slightly wide and runs through the center (Sample 5), all models except the traditional model correctly identified the crack.

### 5.3. Multi-Class Image Classification Metrics

**Figure 17:**
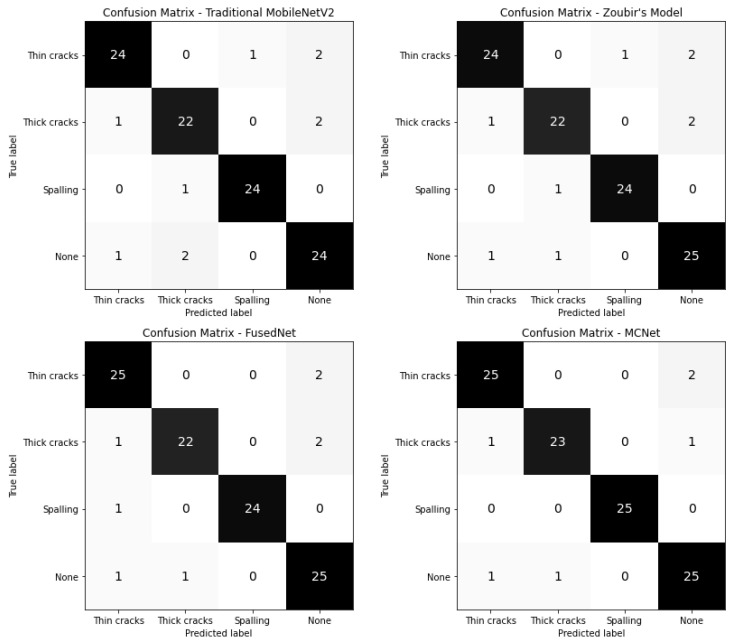
Confusion matrices of traditional MobileNetV2, Zoubir, FusedNet, and MCNet models for multi-class image classification.

**Figure 18:**
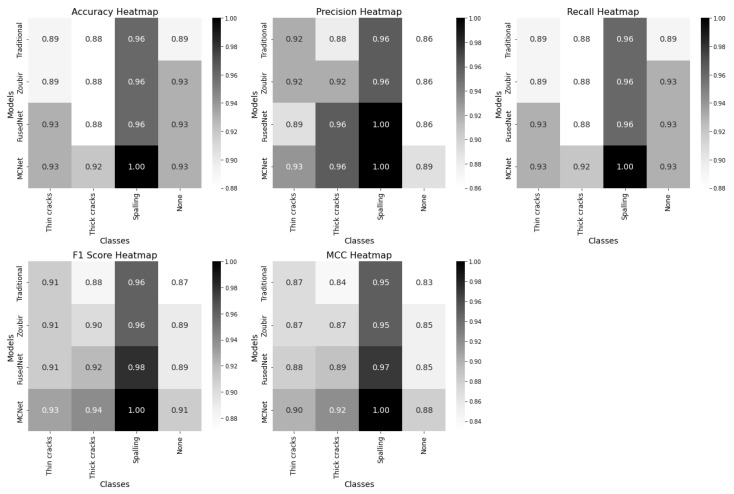
Heatmaps for traditional MobileNetV2 vs. Zoubir vs. FusedNet vs. MCNet models for multi-class image classification.

This section compares four models used for multi-class image classification tasks—Traditional MobileNetV2, Zoubir’s Model, FusedNet, and MCNet—across several performance metrics: accuracy, precision, recall, F1 score, and Matthews Correlation Coefficient (MCC). Each model’s performance is visualized through confusion matrices and heatmaps shown in Figure 17 and Figure 18.

The confusion matrices provide an overview of how each model classifies instances of thin cracks, thick cracks, spalling, and none. This visualization highlights the number of true positives, false positives, false negatives, and true negatives for each class, giving insight into the classification behavior of the models.

Each heatmap row represent the models—Traditional MobileNetV2, Zoubir’s model, FusedNet, and MCNet—while the columns correspond to the target classes: thin cracks, thick cracks, spalling, and none. The color shades in each cell reflect the magnitude of the metrics, with darker shades indicating lower values and lighter shades representing higher values.

In the accuracy heatmap, MCNet and FusedNet demonstrated similar performance for detecting thin cracks. However, MCNet excelled in detecting thick cracks, and achieved the highest accuracy of 92%. Notably, MCNet achieved 100% accuracy in identifying spalling. FusedNet outperformed both the traditional model and Zoubir’s model in detecting thin cracks. The performance of all models was comparable for detecting thick cracks and spalling. However, FusedNet consistently performed better than the traditional model and Zoubir’s model. Zoubir’s model and the traditional model exhibited similar metrics, with the traditional model slightly trailing behind.

In terms of precision, both MCNet and FusedNet exhibited superior performance in detecting thick cracks, spalling, and the absence of defects compared to the traditional model and Zoubir’s model. For thin cracks, MCNet achieved an identical precision of 93%, while FusedNet is slightly lower at 89%. Zoubir’s model falls between the traditional and the proposed models, showing intermediate precision values.

The recall heatmap demonstrates that MCNet achieves the highest recall across most defect categories, indicating its effectiveness in identifying true positives and minimizing missed defects. MCNet and FusedNet both outperformed the traditional model and Zoubir’s model in detecting thin and thick cracks, with MCNet showing exceptional recall for spalling. FusedNet also shows high recall values but slightly lags behind MCNet. Zoubir’s model, while performing well, does not match the proposed models in overall recall. FusedNet also performed competitively, particularly for thick cracks and spalling, but with slightly lower scores compared to MCNet. Both the proposed models have shown better F1 scores than the three-channel models.

The MCC heatmap indicates that MCNet achieves the highest MCC scores across most defect categories, highlighting its robustness in predicting both true positives and true negatives while minimizing false positives and false negatives. FusedNet also performed well, especially for thick cracks and spalling, but slightly trails behind MCNet. The traditional model and Zoubir’s model have shown moderate MCC scores, with Zoubir’s model generally positioned between the traditional model and the advanced models.

Across all metrics, MCNet and FusedNet consistently outperformed the traditional model and Zoubir’s model, with MCNet often leading in performance. MCNet is particularly strong in detecting specific types of defects, such as spalling, making it the preferred choice for applications requiring high accuracy in defect detection. For balanced performance across various defect types, both MCNet and FusedNet are suitable, though MCNet holds a slight edge due to its superior performance across multiple metrics. Zoubir’s model performs well but is too general and did not reach the performance scores of the advanced models. This analysis highlights the significant impact of advanced models like MCNet and FusedNet in enhancing the ability to identify and address structural defects effectively in civil engineering applications.

**Figure 19:**
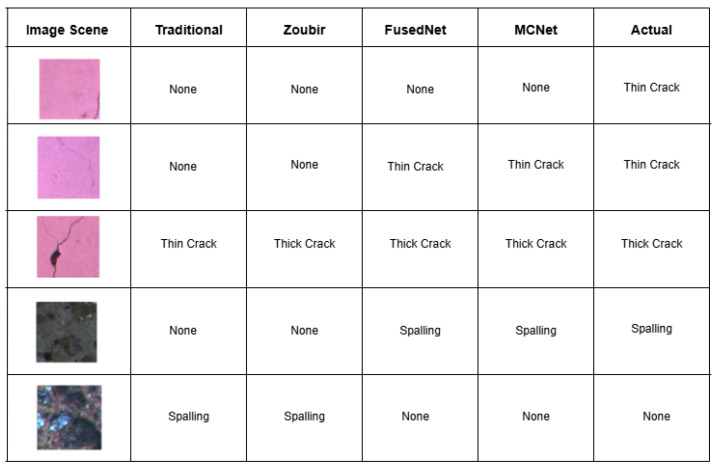
Model comparison for multi-class image classification on various samples.

Figure 19 shows qualitative results obtained when four trained models are tested on various samples to detect cracks and spalling. When the crack is located near the extremities, such as along the edges (Sample 1), all models failed to detect it. Both proposed models detected the hairline crack passing almost through the center (Sample 2). When the crack is thick and passes through the center (Sample 3), both proposed models and Zoubir’s model correctly detected it as a thick crack, unlike the traditional model, which misclassified it as a thin crack.

Both the proposed models identified spalling in Sample 4. The traditional and Zoubir’s models mistakenly identified Sample 5 as spalling. Overall, it is evident that MCNet outperforms the other three models in detecting both cracks and spalling. This superior performance is due to the light being projected onto the surface from all directions, which enhances the visibility of crack extremities and thereby improves defect detection in civil engineering applications.

### 5.4. Effect of Exposure on Diffused and Fused Images

#### 5.4.1. Analyze the Performance of Diffused Images Captured Under Different Exposure Values

The traditional MobileNetV2 model uses diffused images, while FusedNN creates a fused image by selecting the maximum intensity pixel values from the directional images. So far, the analysis has been conducted under auto exposure settings.

If the images are captured under increased exposure conditions, the resulting images will display brighter pixels. Since the FusedNN model selects the maximum intensity pixel values, the pixels from the directional images will also have high exposure values. At a certain point, even though FusedNN selects the maximum intensity pixels, the fused image and the diffused image could become identical due to the uniformly high exposure. If this occurs, the advantage of using FusedNN diminishes, as there would be no significant difference between the fused image and the diffused image. Thus, the potential benefit of FusedNN in enhancing defect detection would be negated under such high exposure conditions.

This scenario highlights the importance of considering exposure settings in evaluating the true efficiency of FusedNN compared to traditional methods. For this reason, an evaluation was conducted to compare the performance of the traditional MobileNetV2 and FusedNN models under different exposure conditions. Various performance metrics were utilized to assess the performance of the model, including accuracy, precision, recall, F1 score, and MCC.

Images of two concrete slabs were captured using auto exposure settings, starting with an initial value of 470,000 lux-seconds. To create underexposed and overexposed conditions, the exposure value was adjusted between 200,000 and 900,000 lux-seconds. This process was repeated to capture directional images (R, D, L, U), all at an optimal angle of 50 degrees. A stratified five-fold cross-validation technique was then employed for binary classification of these images, training and testing the model on datasets with exposure values ranging from 200,000 to 900,000 lux-seconds. The objective was to identify the optimal exposure value.

The results of the evaluation as shown in Table 6 show that the traditional MobileNet model consistently achieved high accuracy across different exposure values, ranging from 89.3% to 92.6%, with the highest accuracy of 92.6% being achieved at an exposure value of 600,000 lux-seconds. The F1 scores, representing the harmonic mean of precision and recall, followed a similar trend, with the highest F1 score of 92.5% observed at the optimal exposure value of 600,000 lux-seconds. Moreover, the MCC values, assessing the quality of binary classifications, maintained a consistent pattern, with the peak MCC of 85.5% achieved at an exposure value of 600,000 lux-seconds. Based on these performance metrics, it can be concluded that an exposure value of 600,000 lux-seconds is the optimal setting for this model when utilizing diffused images. The ability of the model to differentiate between cracked and uncracked samples under different lighting conditions is highlighted by its strong performance at this exposure value.

#### 5.4.2. Evaluation of Fused and Diffused Images

After obtaining the optimal exposure value, the aim was to explore the potential of fused images. To achieve this, images were captured under different lighting directions, each sharing similar exposure levels. These values were as follows:

- Right Direction: 1.63 million lux-seconds;
- Down Direction: 1.65 million lux-seconds;
- Left Direction: 1.68 million lux-seconds;
- Up Direction: 1.71 million lux-seconds.

**Figure 20:**
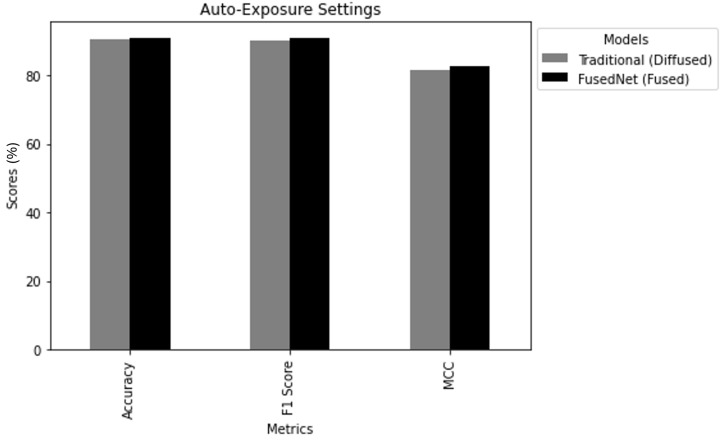
Comparison of model performance in auto-exposure settings.

These images were combined with the diffused image captured at the optimal exposure value of 600,000 lux-seconds to create a fused image. The main objective was to evaluate the performance of these fused images against the diffused image and to study the effect of increased exposure on the performance of the model. The research comprised two distinctive scenarios to assess the performance of the model:

i. Auto-Exposure Comparison: In this scenario, traditional MobileNetV2 and FusedNN models were trained and tested using the auto-exposure diffused image and the auto-exposure fused image, respectively. The aim was to understand how well these models performed using images captured at their auto-exposure values. Figure 20 draws the following conclusions:

- Accuracy: FusedNN achieved an accuracy of 91.1%, exceeding the traditional model’s accuracy of 90.4%.
- F1Score: The F1 score was 91% FusedNN, exceeding traditional model’s F1 score of 90.24%.
- MCC: Finally, FusedNN achieved a higher MCC of 82.7% compared to the traditional model’s MCC of 81.77%.

**Figure 21:**
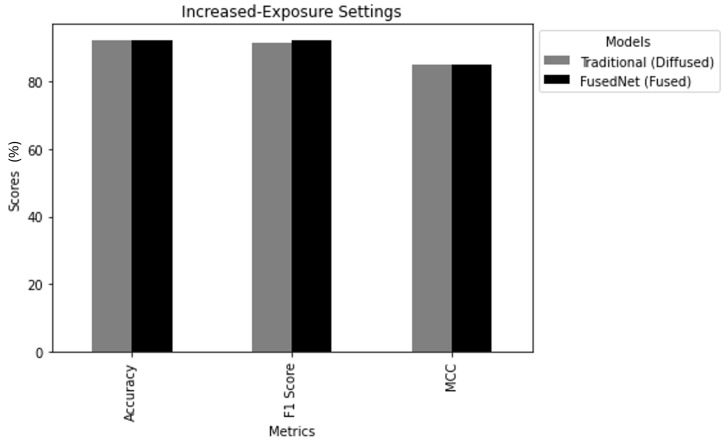
Comparison of model performance in increased-exposure settings.

This indicates that FusedNN had a superior ability to make accurate binary classifications under auto-exposure conditions.

ii. Increased Exposure Comparison: The second scenario focused on the impact of increased exposure on the performance of the model. Here, the performance of the MobileNetV2 and FusedNN models was evaluated when trained and tested on diffused and fused images, respectively, under increased exposure conditions. The aim is to understand how increased exposure levels affect the performance of the model for crack classification. Figure 21 draws the following conclusions:

- Accuracy: The traditional model achieved an accuracy of 92.1%, while FusedNN reached an accuracy of 92.22%. Both models performed well, with FusedNN maintaining a slight edge.
- F1Score: The F1 score reflects the balance between precision and recall. The traditional model achieved an F1 score of 91.53, while FusedNN obtained a high F1 score of 92.3.
- Matthews Correlation Coefficient: The traditional model reached an MCC of 85, while FusedNN achieved an MCC of 85, indicating strong binary classification capabilities.

In the increased exposure scenario, FusedNN still demonstrated superior performance compared to the traditional MobileNetV2 model. This suggests that FusedNN is more reliable and robust in accurately identifying cracked samples, regardless of the exposure conditions.

The study showed that the FusedNN model using fused images (directional images) was effective in auto-exposure and increased exposure settings compared to the traditional model using diffused images alone. This highlights the adaptability and robustness of the fused image approach. This indicates that the dependence of the FusedNN model on maximum-intensity pixel values remains effective under varying exposure settings. In conclusion, this study highlights the potential of the fused image technique, which outperforms traditional diffused images and maintains its effectiveness under modified exposure levels.

**Figure 22:**
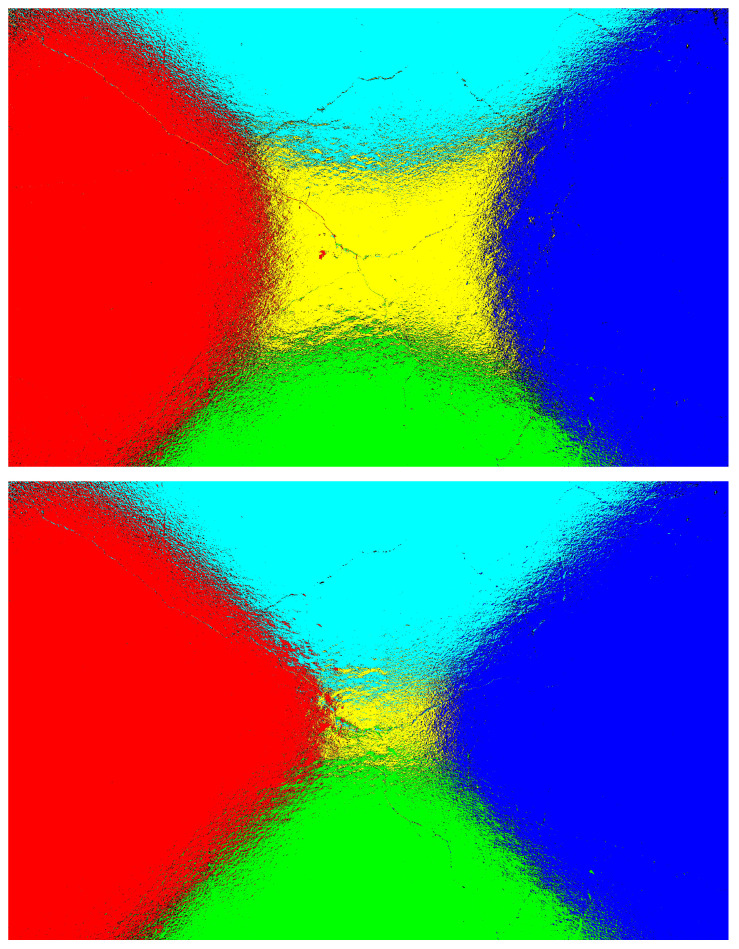
Sample 1: Comparison of fused images generated using directional images captured at auto-exposure and increased-exposure settings.

**Figure 23:**
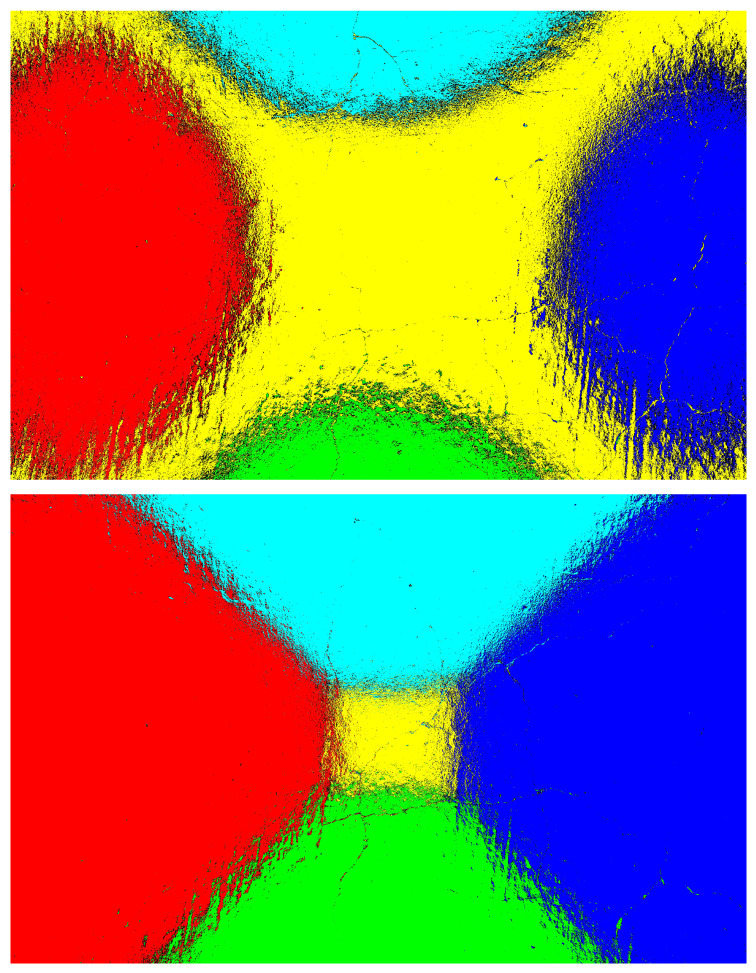
Sample 2: Comparison of fused images generated using directional images captured at auto-exposure and increased-exposure settings.

Figure 22 and Figure 23 represent the heatmaps of the fused images generated using images captured at auto-exposure and increased-exposure settings for samples-1 and 2. Even with higher exposure levels, the fused image still depended on selecting the maximum intensity pixels from the directional images. This approach helped in extracting valuable information from varying lighting conditions, leading to enhanced visibility of cracks and an improved classification rate, as shown in Figure 20 and Figure 21.

#### 5.4.3. Comparative Analysis of Traditional MobileNetV2 and FusedNN Models Under Different Exposure Settings

**Figure 24:**
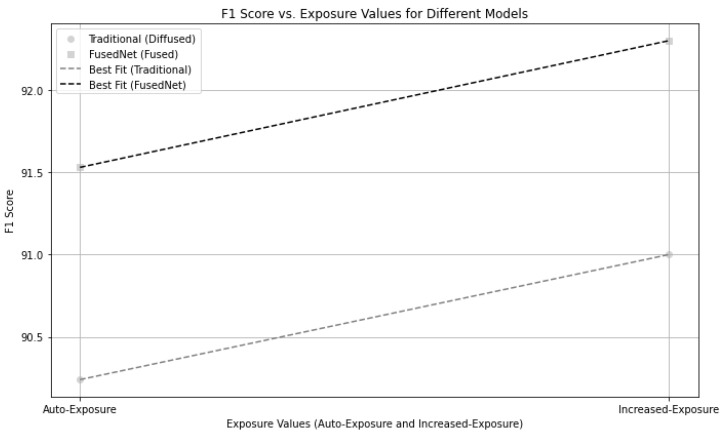
Comparative Performance of traditional MobileNetV2 and FusedNN models under auto and increased exposure settings.

The comparative analysis of the Traditional MobileNetV2 and FusedNN models, as shown in Figure 24, clearly demonstrates the advantage of FusedNN over the Traditional MobileNetV2 model. The X-axis represents various exposure values, including both auto-exposure and increased exposure settings, along with the corresponding F1 scores on the Y-axis for both models. The best fit lines indicate the trend in their F1 scores, indicating that as the exposure value increases, the performance of both models also increases. However, FusedNN consistently outperformed Traditional MobileNetV2 across all exposure settings.

The improved performance of FusedNN is due to its novel approach of selecting the maximum intensity pixels, which proves to be advantageous compared to traditional MobileNetV2 model. In summary, increasing the exposure improves the performance of vision models, particularly in terms of the F1 score. However, FusedNN outperforms Traditional MobileNetV2 as a more efficient model across different exposure settings. This makes FusedNN an ideal choice for a wide range of applications in the field of civil engineering.

#### 5.4.4. Examining the Effects of Auto-Exposure on Machine Vision

Auto-exposure is an algorithm that creates brighter images by adapting camera settings like aperture, shutter speed, and ISO. Generated images are designed with human viewers in mind, which can sometimes lead to conflict with machine vision systems’ need for accurate image data. Under sub-optimal lighting conditions—including low light, uneven light, and backlighting—auto-exposure can cause severe distortions and degrade the image quality. For example, uneven lighting can result in both overexposed and underexposed regions, impacting human visual perception as well as image recognition capabilities. In the case of high-contrast scenes, auto-exposure algorithms often face challenges as they struggle or try to achieve a balanced exposure throughout the image. This can lead to overexposed shadows and underexposed bright areas. Such imbalance can be harmful when trying to detect features such as cracks in concrete structures [78]. FusedNN, by utilizing maximum-intensity pixels in fused images, exceeds auto-exposure algorithms in multiple scenarios. This method maintains the ability of the model to detect fine details, even in challenging lighting environments.

#### 5.4.5. Advantages of Fused Image Technique Under Varying Exposure Conditions

The findings highlight the strong performance and reliability of the FusedNN model utilizing the fused image technique when faced with varying exposure conditions. By utilizing maximum-intensity pixel values, FusedNN is able to effectively handle different levels of exposure while maintaining high accuracy in distinguishing between cracked and uncracked samples. This approach guarantees that crucial crack details are preserved, ensuring accurate identification of cracks. The adaptability of the fused image technique is further highlighted in these findings. It efficiently handles variations in lighting conditions, demonstrating that FusedNN maintains its effectiveness even when exposed to different illumination levels. This highlights the potential of the fused image technique, exceeding traditional diffused images under various exposure settings.

### 5.5. Evaluation Time and Model Size

The evaluation times for the Zoubir and MCNet models highlight a significant advantage of MCNet in terms of processing speed. The Zoubir’s model requires 87 milliseconds to evaluate an image, whereas MCNet only needs 11 milliseconds, making MCNet approximately eight times faster. Such faster evaluation times are crucial for tasks like concrete structure inspections, where timely and accurate crack detection is essential for maintaining structural integrity and safety. Also, the traditional MobileNetV2 model needs 10 milliseconds to evaluate an image, showing that MCNet’s additional two channels do not significantly impact evaluation time.

The difference in parameter count between the three-channel MobileNetV2 and the MCNet comes entirely from the first convolutional layer, where the change from three to five channels adds only 576 parameters (see Table 3). This increase is negligible relative to the total model size. These results confirm that MCNet achieves a substantial speed advantage, and remains lightweight enough for real-time deployment in field inspections of concrete structures.

The MCNet architecture implemented in this study was configured for a five-channel input, corresponding to the five lighting directions used during data acquisition. While the current implementation operates with exactly five channels, the design is inherently adaptable to any number of input channels, which is reflected in the name multi-channel neural network (MCNet) model. If the number of lighting directions were to be increased or decreased, the architecture could be modified accordingly by adjusting the first convolutional layer to accept an n-channel input.

### 5.6. Importance of Directional-Lighting and Multi-Channel Input

Directional lighting in the FusedNet and MCNet models played a crucial role in enhancing the accuracy, precision, recall, F1 score, and MCC of defect detection in concrete structures. By capturing images under different lighting conditions, these models were able to extract more information, resulting in more focused and accurate defect detection, particularly in low-light environments. The directional lighting and multi-channel input utilized in MCNet significantly contributed to its superior performance. By capturing information from different lighting directions, MCNet effectively detected and classified defects even in low-light environments, allowing the model to handle variations in patterned lighting and enhancing defect detection accuracy in concrete structures.

Although FusedNet displayed strong performance, MCNet demonstrated superior capabilities, highlighting the significance of incorporating multi-channel input and directional lighting in image classification tasks for concrete defect detection. The findings of this study emphasized the importance of advanced models like MCNet and the adoption of directional lighting techniques in achieving accurate and efficient defect detection and classification in concrete structures. These approaches offer promising solutions for improving infrastructure inspection and maintenance processes. By evaluating the models on diverse data subsets and using stratified five-fold cross-validation and hyperparameter tuning, we reduced the risk of bias and overfitting and validated the models’ generalization capabilities. For the MCNet model, modifications were made to the first convolutional layer to accommodate the five-channel input, allowing the model to efficiently learn relevant features from the new dataset, resulting in accurate defect detection.

### 5.7. Balanced Evaluation

In this section, we outlined our methodology for comparing the performance of the traditional three-channel model, the Zoubir model, the FusedNet model, and the MCNet model. We aimed to ensure a fair comparison by addressing various factors as explained below:

One of the advantages of using multiple directions for image capture in FusedNet and MCNet is the availability of additional information that can aid in defect detection. These models proved to perform better in challenging lighting conditions and enhanced accuracy due to their ability to fuse data from various directions. To maintain consistency across the models, we rigorously ensured that the same images were used in the training and testing datasets for all four models. This step is crucial in ensuring that any performance differences observed are primarily due to architectural variations rather than data variations.

Three different learning rates (0.1, 0.01, and 0.001) and batch sizes of 16, 32, and 64 were considered. Each model was tested with various combinations of these parameters, and the optimal combination was selected to prevent overfitting and ensure that the model generalizes well to new, unseen data. A stratified five-fold cross-validation technique is employed to assess model performance. This approach validates the models’ abilities and indicates their generalization to new, unseen data.

### 5.8. Limitations and Future Work

Extensive measures are taken to ensure a fair comparison, but it is essential to acknowledge certain limitations in our study. Although FusedNet and MCNet models demonstrated superior performance, they are more complex than traditional three-channel and Zoubir’s models due to their use of directional images to generate fused and five-channel images.

From an operational standpoint, capturing images under five lighting conditions introduces significant time and cost overheads. It requires hardware modifications (e.g., positioning equipment for different directions), which add procurement, installation, and maintenance costs. Directional images require five times the capturing time compared to diffused images. As computational time increases, so do the expenses related to hardware resources and electricity consumption, thereby raising computational costs. Additionally, handling this hardware becomes challenging under adverse weather conditions, such as heavy rainfall and strong winds, necessitating a more robust version.

The traditional models and Zoubir’s model use a single diffused image, while the FusedNet and MCNet models consider images from five different directions. This introduces a fundamental difference in the input data. Although the comparison in this study has limitations, it shows that directional lighting can improve accuracy in real-world scenarios. The new models typically have larger dataset sizes, leading to increased storage requirements. This can be a concern when deploying these models on resource-constrained devices. While the models excel in challenging lighting conditions, they are not tested under adverse weather conditions, which could be an area for future investigation.

Future research will focus on expanding these concepts in identifying efflorescence, corrosion, and delamination in concrete structures. Additionally, by leveraging more powerful hardware, such as graphics processing units, we can further enhance the performance of the model in developing automated systems for infrastructure assessment and defect detection in civil engineering. Our study found that the FusedNet and MCNet models significantly outperformed the traditional and Zoubir’s models based on accuracy, precision, recall, F1 score, and MCC. We made sure to compare the models fairly and discussed their limitations, which makes our results reliable and can help future research in this field.

## 6. Conclusions

This paper presented two methods for detecting concrete spalling and cracking using directional lighting. The methods, FusedNet and MCNet, both utilized multiple images, each illuminated under lighting projected from the right, down, left, up, and diffused directions. The aim was not to develop a model that is state-of-the-art. The focus was to improve crack inspections in concrete structures and further existing technologies in civil engineering using directional lighting.

Stratified five-fold cross-validation, hyperparameter tuning, and regularization techniques were utilized to avoid overfitting and generalize well on new and unseen data. These models are evaluated both on laboratory datasets and real-world datasets. In binary crack classification, MCNet outperformed FusedNet by 3.6%, Zoubir’s model by 4.5%, and the traditional model by 5%. Similarly, for multi class image classification, MCNet outperformed all the models and achieved 93%, 92%, and 100% accuracy in detecting thin cracks, thick cracks, and spalling, respectively. The proposed models are capable of detecting cracks of width ranging from 0.07 mm to 0.3 mm. Therefore, these models offer promising implications for automated inspection systems in real-world scenarios and contribute to safer and more cost-effective maintenance practices. The improved performance of the proposed models has been achieved with no significant change in evaluation time. Future research involves extending the concepts of multi-channel and image fusion to white-box techniques and analyzing their performance in various environmental conditions.

## Figures and Tables

**Table 1:** Bottleneck of MobileNetV2 taken from [61].

Input	Operator	Output
H × W × N	1 × 1 conv2d, ReLU6	H × W × tN
H × W × tN	3 × 3 dwise s = s, ReLU6	H/s × W/s × tN
H/s × W/s × tN	linear 1 × 1 conv2d	H/s × W/s × M

**Table 2:** The overall network structure of MobileNetV2 taken from [61].

Input Shape	Operator	t	c	n	s
224 × 224 × 3	conv2d	-	32	1	2
112 × 112 × 32	bottleneck	1	16	1	1
112 × 112 × 16	bottleneck	6	24	2	2
56 × 56 × 24	bottleneck	6	32	3	2
28 × 28 × 32	bottleneck	6	64	4	2
14 × 14 × 64	bottleneck	6	96	3	1
14 × 14 × 96	bottleneck	6	160	3	2
7 × 7 × 160	bottleneck	6	320	1	1
7 × 7 × 320	conv2d 1 × 1	-	1280	1	1
7 × 7 × 1280	avgpool 7 × 7	-	-	1	-
1 × 1 × 1280	conv2d 1 × 1	-	k	-	

**Table 3:** Parameter comparison: 3-channel vs. 5-channel MobileNetV2 models in the first convolutional layer.

Parameter	Values for Three-Channel MobileNetV2	Values for Five-Channel MobileNetV2
Number of filters	32	32
Filter size	3 × 3	3 × 3
Number of weights per filter	9	9
Total weights for all filters	32 × 9 = 288	32 × 9 = 288
Number of channels	3	5
Total weights for all channels	288 × 3 = 864	288 × 5 = 1440
Total trainable parameters	864	1440

**Table 4:** Performance metrics of a binary classifier.

Name	Description	Formulae
Recall	The proportion of true positive predictions out of all actual positive instances.	$\frac{TP}{TP+FN}$
Precision	The proportion of true positive predictions out of all positive predictions.	$\frac{TP}{TP+FP}$
Accuracy	The measure of the model’s ability to correctly predict the outcomes.	$\frac{TP+TN}{TP+FN+FP+TN}$
MCC	The measure of the correlation between predicted and actual labels, accounting for true or false, both positive and negative predictions.	$\frac{TP\times TN-FP\times FN}{\sqrt{\left(TP+FP)\cdot (TP+FN)\cdot (TN+FP)\cdot (TN+FN\right)}}$
F1 Score	The harmonic mean of precision and recall.	$\frac{2\times \mathrm{Precision}\times \mathrm{Recall}}{\mathrm{Precision}+\mathrm{Recall}}$

**Table 5:** Performance metrics of a multi-class classification, where i ranges from 1 to the total number of classes, l. The average of the metric across all the classes is computed to obtain the macro average metric. M represents macro averaging. β controls the balance between precision and recall.

Metric	Description	Formulae
$Accuracy_{M}$	The accuracy of each class is computed separately, and then take the average of all the accuracies to obtain macro-averaged accuracy.	$\sum_{i=1}^{l}\frac{1}{l}\frac{TP_{i}+TN_{i}}{TP_{i}+TN_{i}+FP_{i}+FN_{i}}$
$Precision_{M}$	The precision of each class is computed separately, and then take the average of all the precisions to obtain macro-averaged precision.	$\sum_{i=1}^{l}\frac{1}{l}\frac{TP_{i}}{TP_{i}+FP_{i}}$
$Recall_{M}$	The recall of each class is computed separately, and then take the average of all the recalls to obtain macro-averaged recall.	$\sum_{i=1}^{l}\frac{1}{l}\frac{TP_{i}}{TP_{i}+FN_{i}}$
$MCC_{M}$	The MCC of each class is computed separately, and then take the average of all the MCCs to obtain macro-averaged MCC.	$\sum_{i=1}^{l}\frac{1}{l}\frac{TP_{i}\times TN_{i}-FP_{i}\times FN_{i}}{\sqrt{\left(TP_{i}+FP_{i}\right)\times \left(TP_{i}+FN_{i}\right)\times \left(TN_{i}+FP_{i}\right)\times \left(TN_{i}+FN_{i}\right)}}$
$F1{-\mathrm{Score}}_{M}$	The F1 score of each class is computed separately, and then the average of all the F1 scores is taken to obtain the macro-averaged F1 score.	$\frac{\left(1+\beta^{2}\right)\times {\mathrm{Precision}}_{M}\times {\mathrm{Recall}}_{M}}{\left(\beta^{2}\times {\mathrm{Precision}}_{M}\right)+{\mathrm{Recall}}_{M}}$

**Table 6:** Performance metrics at different exposure levels.

Exposure Values (in Lux-Seconds)	Accuracy	F1 Score	MCC
200,000	89.3	88.8	79.4
300,000	90.6	90	81.6
400,000	92	91.9	84.4
470,000 (Auto-Exposure)	92.36	92	85
500,000	92.5	92.2	85.4
600,000	92.6	92.5	85.5
700,000	92.2	91.83	84.9
800,000	91.53	91.3	83.6
900,000	90.3	89.5	81.2

## Data Availability

The data that has been used is confidential.

## Abbreviations

The following abbreviations are used in this manuscript:

CNN	Convolutional Neural Network
DoF	Depth of Field
FC	Fully Connected
FFT	Fast Fourier Transform
FN	False Negative
FP	False Positive
FusedNet	Fused Neural Network
MCC	Matthew’s Correlation Coefficient
MCNet	Multi-Channel Neural Network
NDE	Non-Destructive Evaluation
NDT	Non-Destructive Testing
RCNN	Region-Based Convolutional Neural Network
ReLU	Rectified Linear Unit
RGB	Red–Green–Blue
SHM	Structural Health Monitoring
SKCV	Stratified k-Fold Cross-Validation
TIFF	Tag Image File Format
TN	True Negative
TP	True Positive
VGG	Visual Geometry Group
YOLO	You Only Look Once
